# A new G-quadruplex-specific photosensitizer inducing genome instability in cancer cells by triggering oxidative DNA damage and impeding replication fork progression

**DOI:** 10.1093/nar/gkad365

**Published:** 2023-05-16

**Authors:** Marco Deiana, José María Andrés Castán, Pierre Josse, Abraha Kahsay, Darío Puchán Sánchez, Korentin Morice, Natacha Gillet, Ranjitha Ravindranath, Ankit Kumar Patel, Pallabi Sengupta, Ikenna Obi, Eva Rodriguez-Marquez, Lhoussain Khrouz, Elise Dumont, Laura Abad Galán, Magali Allain, Bright Walker, Hyun Seo Ahn, Olivier Maury, Philippe Blanchard, Tangui Le Bahers, Daniel Öhlund, Jonas von Hofsten, Cyrille Monnereau, Clément Cabanetos, Nasim Sabouri

**Affiliations:** Department of Medical Biochemistry and Biophysics, Umeå University, SE-901 87, Umeå, Sweden; Univ Angers, CNRS, MOLTECH-ANJOU, SFR MATRIX, F-49000 Angers, France; Univ Angers, CNRS, MOLTECH-ANJOU, SFR MATRIX, F-49000 Angers, France; Department of Integrative Medical Biology, Umeå University, SE-901 87, Umeå, Sweden; Univ Angers, CNRS, MOLTECH-ANJOU, SFR MATRIX, F-49000 Angers, France; Univ Angers, CNRS, MOLTECH-ANJOU, SFR MATRIX, F-49000 Angers, France; ENS de Lyon, CNRS, Université Claude Bernard Lyon 1, Laboratoire de Chimie UMR 5182, F-69342 Lyon, France; ENS de Lyon, CNRS, Université Claude Bernard Lyon 1, Laboratoire de Chimie UMR 5182, F-69342 Lyon, France; Indian Institute for Science Education and Research (IISER), Tirupati-517507, India; Department of Radiation Sciences/Oncology, Umeå University, SE-901 87, Umeå, Sweden; Wallenberg Centre for Molecular Medicine (WCMM), Umeå University, SE-901 87, Umeå, Sweden; Department of Medical Biochemistry and Biophysics, Umeå University, SE-901 87, Umeå, Sweden; Department of Medical Biochemistry and Biophysics, Umeå University, SE-901 87, Umeå, Sweden; Department of Integrative Medical Biology, Umeå University, SE-901 87, Umeå, Sweden; ENS de Lyon, CNRS, Université Claude Bernard Lyon 1, Laboratoire de Chimie UMR 5182, F-69342 Lyon, France; ENS de Lyon, CNRS, Université Claude Bernard Lyon 1, Laboratoire de Chimie UMR 5182, F-69342 Lyon, France; Institut Universitaire de France, 5 rue Descartes, 75005 Paris, France; ENS de Lyon, CNRS, Université Claude Bernard Lyon 1, Laboratoire de Chimie UMR 5182, F-69342 Lyon, France; Univ Angers, CNRS, MOLTECH-ANJOU, SFR MATRIX, F-49000 Angers, France; Department of Chemistry, Kyung Hee University, Seoul, 02447, South Korea; Yonsei University, 50 Yonsei-ro, Seodaemun-gu, Seoul, South Korea; ENS de Lyon, CNRS, Université Claude Bernard Lyon 1, Laboratoire de Chimie UMR 5182, F-69342 Lyon, France; Univ Angers, CNRS, MOLTECH-ANJOU, SFR MATRIX, F-49000 Angers, France; ENS de Lyon, CNRS, Université Claude Bernard Lyon 1, Laboratoire de Chimie UMR 5182, F-69342 Lyon, France; Institut Universitaire de France, 5 rue Descartes, 75005 Paris, France; Department of Radiation Sciences/Oncology, Umeå University, SE-901 87, Umeå, Sweden; Wallenberg Centre for Molecular Medicine (WCMM), Umeå University, SE-901 87, Umeå, Sweden; Department of Integrative Medical Biology, Umeå University, SE-901 87, Umeå, Sweden; ENS de Lyon, CNRS, Université Claude Bernard Lyon 1, Laboratoire de Chimie UMR 5182, F-69342 Lyon, France; Univ Angers, CNRS, MOLTECH-ANJOU, SFR MATRIX, F-49000 Angers, France; Yonsei University, 50 Yonsei-ro, Seodaemun-gu, Seoul, South Korea; Building Blocks for FUture Electronics Laboratory (2BFUEL), IRL CNRS 2002, Yonsei University, Seoul, South Korea; Department of Medical Biochemistry and Biophysics, Umeå University, SE-901 87, Umeå, Sweden

## Abstract

Photodynamic therapy (PDT) ideally relies on the administration, selective accumulation and photoactivation of a photosensitizer (PS) into diseased tissues. In this context, we report a new heavy-atom-free fluorescent G-quadruplex (G4) DNA-binding PS, named **DBI**. We reveal by fluorescence microscopy that **DBI** preferentially localizes in intraluminal vesicles (ILVs), precursors of exosomes, which are key components of cancer cell proliferation. Moreover, purified exosomal DNA was recognized by a G4-specific antibody, thus highlighting the presence of such G4-forming sequences in the vesicles. Despite the absence of fluorescence signal from **DBI** in nuclei, light-irradiated **DBI**-treated cells generated reactive oxygen species (ROS), triggering a 3-fold increase of nuclear G4 foci, slowing fork progression and elevated levels of both DNA base damage, 8-oxoguanine, and double-stranded DNA breaks. Consequently, **DBI** was found to exert significant phototoxic effects (at nanomolar scale) toward cancer cell lines and tumor organoids. Furthermore, *in vivo* testing reveals that photoactivation of **DBI** induces not only G4 formation and DNA damage but also apoptosis in zebrafish, specifically in the area where DBI had accumulated. Collectively, this approach shows significant promise for image-guided PDT.

## Graphical Abstract

**Graphical Abstract:**
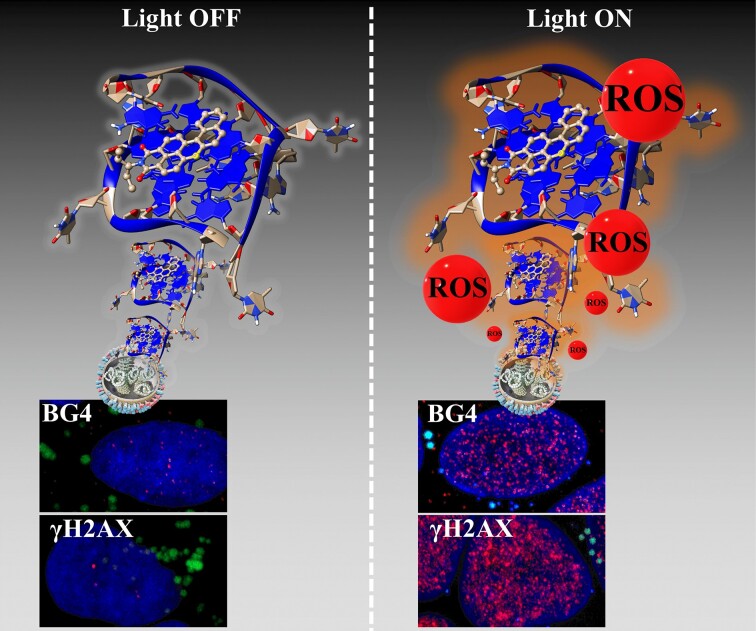
Photoactivation of a new heavy-atom free fluorescent G-quadruplex DNA-binding photosensitizer generates reactive oxygen species (ROS) that results in elevated levels of DNA damage och G-quadruplex formation.

## INTRODUCTION

Pharmacotherapy for tumor ablation is often hindered by poor drug selectivity towards malignant cells, raising safety concerns (1). Photodynamic therapy (PDT) is a light-triggered non-surgical anticancer modality that is used in clinics worldwide (2). PDT involves the use of a photosensitizing agent (PS) that generates cytotoxic reactive oxygen species (ROS) through energy transfer upon exposure to low light power density. The catalytic nature of PDT provides high therapeutic efficacy at low doses and does not generally show cross-resistance to chemotherapy (3). The primary selectivity of PDT comes from the controlled volume of illumination of targeted regions, such as tumors, which can be achieved with high spatiotemporal precision. The type of PS used provides an additional level of selectivity to target specific cancer-related tumor signatures. Indeed, the continuous development in light delivery technologies (4) coupled with the rational design of new and efficient PSs (5) provide means to influence cancer biology with even more precision.

**Figure 1.**
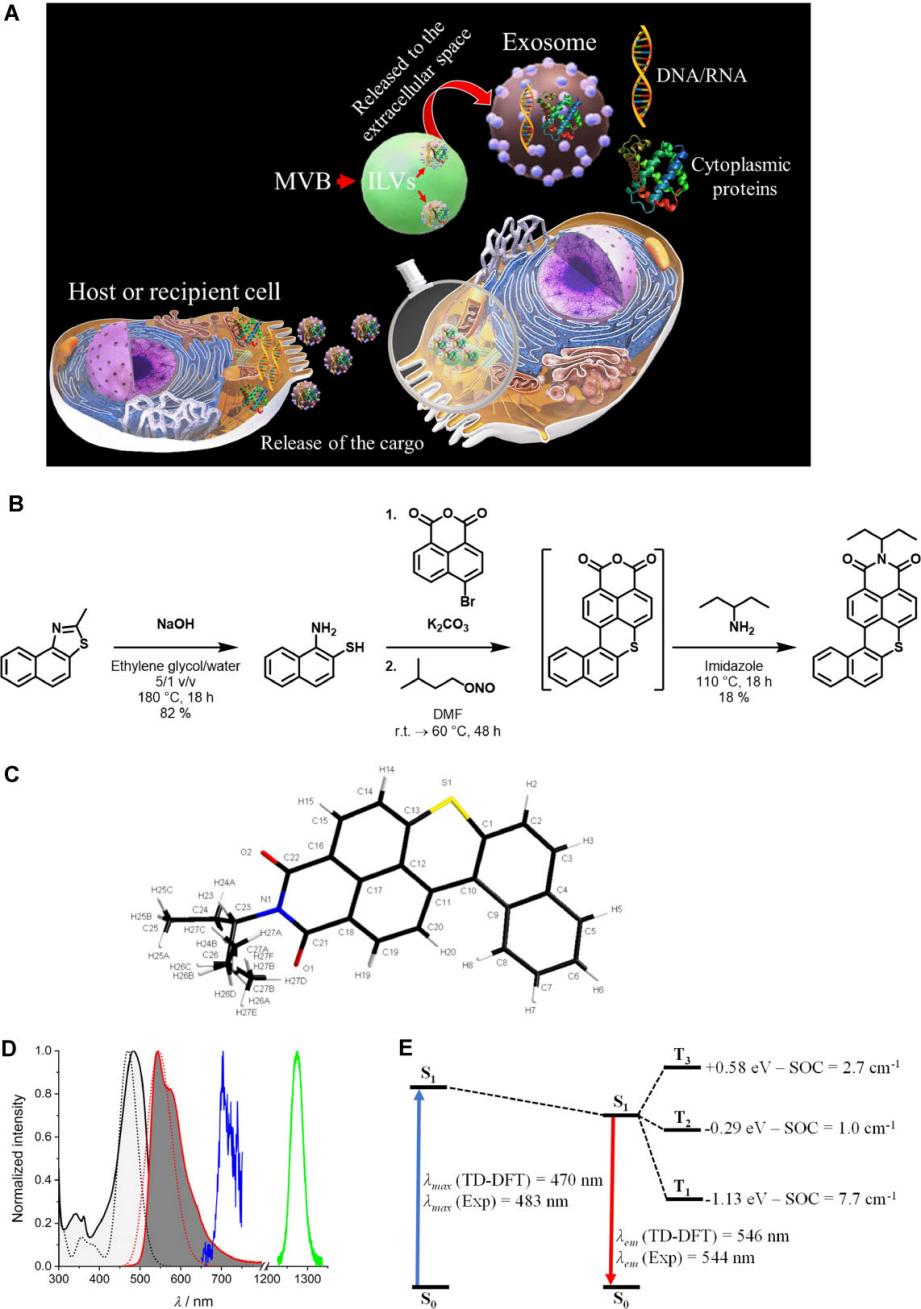
Schematic representation of MVB′s sorting pathways along with the synthetic route of **DBI** and its spectral properties. (**A**) The MVB may fuse with the plasma membrane and release the ILVs as exosomes. Exosomes may epigenetically reprogram recipient cells via delivery of their cargo (DNA, RNA, and proteins). (**B**) Synthesis of **DBI**. (**C**) X-ray crystal structure of **DBI**. (**D**) Experimental (black and red solid lines) and computed (black and red dotted lines) absorption and emission spectral signatures of **DBI** in CH_2_Cl_2_ at 298 K. 77 K time-gated phosphorescence spectrum (blue line, 50 μs delay) and singlet oxygen phosphorescence signal (green line) at 298 K in CH_2_Cl_2_. (**E**) Computed Jablonsky diagram of the **DBI** molecule. Triplet states properties are computed at the S_1_ geometry. The energies of triplet states use the S_1_ energy as reference. The SOC is computed between the triplet states and the S_1_ state.

Heavy-atom-free PSs are a long-sought-after class of chemicals in PDT (6,7). Their excellent photophysical and photochemical properties, along with their low dark toxicity, long-lived triplet excited states, and cost-effective synthesis offer possibilities for the translation of such agents into clinical use in PDT (8). Photoexcitation of the PS promotes the generation of ROS through two mechanisms, namely type I and type II pathways. In the type I pathway, the PS participates in electron transfer reactions to produce radicals (e.g. hydroxyl radical, OH^•^) and radical ions (e.g. superoxide anion, O_2_^•−^) as well as non radical species (e.g. hydrogen peroxide, H_2_O_2_) whereas, in the type II pathway, the PS transfers energy to triplet ground-state molecular oxygen (^3^O_2_), thus generating singlet oxygen (^1^O_2_) (8). These two pathways can happen simultaneously upon light irradiation; yet the type II pathway is the predominant one for most approved PSs (8–10). Indeed, the limited diffusion distance of ^1^O_2_ in cells is an advantage for treatment at restricted sites, while maximizing phototoxic cellular damage using organelle-specific PSs (8,11). In this context, a number of organelle-specific PSs have been reported with affinities to certain organelles such as the nuclei (12–14), mitochondria (15–17), and lysosomes (18,19); their PDT effect has been studied in detail. However, there is to date no PDT strategy that specifically targets multivesicular bodies (MVBs). Rounded or slightly elliptical nanometer-sized (250–1000 nm), the MVBs contain intraluminal vesicles (ILVs) with a typical size of 30–150 nm (20,21). Most of the ILVs, upon fusion of MVBs with the plasma membrane, are released into the extracellular space and are at that time referred to as exosomes (22,23). Exosomes are known to contain a vast array of functionally active biomolecules (DNA, RNA, lipids and proteins) and to be involved in key biological processes including cell-to-cell communication (Figure 1A) (24–27). In particular, exosomes secreted by cancer cells act as the mediator of tumor formation and progression, and often help the cancer cells to escape immune surveillance by inhibiting lymphocyte activation and survival (28). As a putative working mechanism, the direct transfection of altered genetic materials from cancer cells to healthy cells affects the recipients to the point that it may turn the infected cell into a precancerous or cancerous state (29,30). This is supported by the fact that exosome secretion by cancer cells is higher and highly enriched in genomic DNA content compared to healthy cells with approximately 20-fold enrichment (25,31). Consequently, (DNA-rich) tumor-derived exosomes are now emerging as important biomarkers for cancer detection and targeting the latter offers promising possibilities for both monitoring/diagnostic, and therapeutic purposes (21,31).

Genomic DNA is the direct target for most cytotoxic drugs in chemotherapy, which are still extensively used in the treatment of a wide range of human cancers, despite their limited selectivity (32,33). An alternative approach aimed at improving drug selectivity is provided by targeting non-duplex DNA sequences using structure-specific DNA binders (32–35). Guanine (G)-rich DNA sequences can fold into non-canonical four-stranded structures, known as G-quadruplexes (G4s), through the formation of four in-plane G bases mutually bound by Hoogsteen hydrogen bonds and coordinated by metal ions (usually K^+^) (36). The positions of putative G4 structures are not randomly distributed in the nuclear genome but enriched in certain areas such as promoters, ribosomal DNA, and telomeres (36). Moreover, putative G4-forming sequences are also detected in organelles, such as mitochondria (37–39), and have been shown to have a functional role during mitochondrial transcription (40,41). A set of complementary experiments involving the G4-specific antibody (BG4) (42,43), live-cell fluorescent probes (44–46), and chromatin immunoprecipitation followed by high throughput sequencing (ChIP-Seq) (47–50) recently revealed increased levels of G4 structures in cancer cells compared to normal cells. Moreover, G-rich sequences are sensitive to ROS (51), forming oxidatively modified G base lesions, commonly the 8-oxoguanine (8-oxoG) damage. Unrepaired base damages can result in replicative stress and genomic instability (52). Also, modulation of G4 structures using G4-interactive binding ligands can induce replicative stress via interference with DNA transactions, i.e. DNA replication and transcription, leading to single- (SSBs) (53) and double-stranded breaks (DSBs), as well as activation of the DNA damage response (DDR) machinery (54). Thus, the induction of oxidative stress and G4 formation can be applied in cancer therapeutics, as many tumor cells have impaired DDR and/or antioxidant pathways (55–57).

In this context, we introduce herein a novel ‘manifold’ PDT strategy that crucially relies on a heavy-atom free PS, called dibenzothioxanthene imide (**DBI**) that features a twisted *π*-conjugated system providing enhanced intersystem crossing (ISC) capability and consequential high ROS generation efficiency. In particular, **DBI** shows significant phototherapeutic efficacy at nanomolar concentrations against monolayer cancer cells and 3D tumor organoids. *In vivo* experiments with zebrafish embryos further confirmed **DBI**’s PDT effect. Through a set of imaging procedures and biochemical characterizations, we unveil **DBI**′s cellular localization in ILVs/exosomes and its selective interaction to G4 structures. An in-depth investigation of **DBI**′s phototoxic effects led us to discover a close connection between (photo)-induced DNA damage, G4 structures, and replicative stress that led to cell death. We believe that the combination of the aforementioned collective features and the dual targeting of cancer mediators/markers ILVs/exosomes and G4s provides promising opportunities for broad biomedical applications that include, but are not limited to, cancer treatments. Indeed, **DBI**-mediated DNA damage, which strongly differs from the ones reported for G4-stabilizing ligands (54), can be, in principle, modulated by adjusting not only the **DBI** concentration but also the power, illumination time and even the wavelength of the light source. This unique feature provides a means to implement **DBI** as a versatile tool for image-guided photopharmacological applications.

## MATERIALS AND METHODS

Detailed synthetic methods, purification, characterization, spectroscopic and computational procedures involving **DBI** are described in the Supplementary Information. Experimental factors such as the initial number of seeded cells, time of irradiation, and the incubation time of the cells after irradiation were optimized for the different experiment types. These factors are described in more detail in this section and/or in the figure legends.

### Photo-cytotoxicity on 2D monolayer cancer cell lines

HeLa and MCF-7 cells were cultured at 37°C in 5% CO_2_ in DMEM medium supplemented with penicillin-streptomycin (1×), and 10% fetal bovine serum. 5 × 10^3^ cells/well (both HeLa and MCF-7) were seeded in complete medium on 96 well-plates 24 h before the treatment with **DBI**. **DBI** was dissolved in complete medium at the indicated concentrations (DMSO reached the max value of 0.5% v/v) and was added to cells. When required, the cells were photo-irradiated using an EVOS® FL cell imaging system equipped with an adjustable-intensity LED cube (Invitrogen, Ref. No.: AMEP4651; Ex: 470/22 nm) operating at 27 mW cm^−2^ for 6 min. Then, the cells were incubated for an additional 24 h at 37°C in 5% CO_2_. At 48 h after **DBI** treatment, PrestoBlue™ (Invitrogen, Ref. No.: A13261) was added to each well and the cells were incubated at 37°C in 5% CO_2_ for three additional hours. Cell viability was measured by recording the fluorescence signal of PrestoBlue (*λ*_exc_/λ_em_: 560/590 nm) using a Synergy H4 microplate reader (Biotek).

Similar experimental set-up was used with Temoporfin (MedChemExpress, Ref. No.: 122341-38-2) in HeLa cells, either under blue LED (Ex: 470/22 nm) or green LED (Invitrogen, Ref. No.: AMEP4952; Ex: 542/20 nm) illumination operating at 27 mW cm^−2^ or 10 mW cm^−2^ for 6 min, respectively.

**DBI** photo-induced cell death was cross-validated using the LIVE/DEAD™ viability/cytotoxicity kit (Invitrogen, Ref. No.: L34960). Briefly, 20 × 10^4^ cells were seeded the day before treatment on glass-bottom microwell dishes (MatTek Corp.). HeLa cells were treated with **DBI** (1 μM) or with an equivalent amount of DMSO (0.02% v/v) and incubated at 37°C in 5% CO_2_ for 24 h. When required, the cells were photo-irradiated as described above (i.e. 27 mW cm^−2^ for 6 min). Then, the cells were incubated for an additional 24 h at 37°C in 5% CO_2_. 48 h after **DBI** treatment, LIVE/DEAD fixable red stain (1 μl /ml) was added to the cells for 30 min at 37°C before fixation with 4% paraformaldehyde (PFA). Before imaging, the cells were further washed with 1 × PBS supplemented with 1% bovine serum albumin. Images were acquired using a Leica SP8 FALCON confocal microscope. Maximum intensity projection of Z-stack images was used for data presentation. All data were processed by using ImageJ software.

### Light-induced morphological changes on DBI-treated HeLa cells

10 × 10^4^ HeLa cells were seeded in complete medium on glass-bottom microwell dishes (MatTek Corp.) for 48 h at 37°C in 5% CO_2_. Cells were washed with 1 × PBS, and **DBI** (1 μM), Temoporfin (10 μM), or an equivalent amount of DMSO (0.02% v/v), dissolved in complete medium and were added to cells for 1 h at 37°C in 5% CO_2_. Then, the cells, when required, were photo-irradiated using an EVOS® FL cell imaging system equipped with an adjustable-intensity LED cube (Ex: 470/22 nm) operating at 30 mW cm^−2^ at various time intervals.

### Photo-cytotoxicity on 3D multicellular pancreatic tumor organoids

Mouse pancreatic tumor 3D organoids were a gift from David Tuveson (Cold Spring Harbor Laboratory, Cold Spring Harbor, New York). The organoids were established using tumors from KPC (C57Bl/6 *Kras^+/LSL-G12D^*; *Trp53^+/LSL-R172H^*; *Pdx-Cre)* mice using methods developed previously (58). KPC mice rapidly develop spontaneous tumors in the pancreas, genetically and histologically mimicking pancreatic ductal adenocarcinoma (PDA) (59). The organoids were cultured embedded in growth factor reduced basement membrane matrix (Matrigel) and grown in defined growth factor media (58).

To examine the cytotoxic effects of **DBI** on the organoids, different concentrations of **DBI** were added to the cells 16 hours after seeding in duplicate and were allowed to grow for a further 24 h. Only one set of plates were exposed to 470 nm light (GFP channel) for 6 minutes at 27 mW cm^−2^ in an EVOS-FL microscope (light condition) while the other set was continuously maintained in the dark (dark condition). Cells were incubated for a further 24 h and were assayed for cell viability using PrestoBlue® (Thermo Fisher Scientific). Data was recorded using SpectraMax® i3x (Molecular Devices). Organoids isolated from three different KPC mice were used in the experiments (biological replicates).

### Photoinduced cellular generation of ROS

20 × 10^4^ HeLa cells were seeded the day before treatment on glass-bottom microwell dishes (MatTek Corp.). HeLa cells were treated with **DBI** (1 μM) or with an equivalent amount of DMSO (0.02% v/v) and incubated at 37°C in 5% CO_2_ for 24 h. When required, the cells were photo-irradiated using an EVOS® FL cell imaging system equipped with an adjustable-intensity LED cube (Ex: 470/22 nm) operating at 30 mW cm^−2^ for 20 min. Then, CellROX green reagent (Invitrogen, Ref. No.: C10444) (5 μM) was added to the cells for 30 min at 37°C in 5% CO_2_. Washing with 1 × PBS (2 times) was performed before fixation in 4% PFA. Before imaging, the cells were further washed with 1 × PBS (3 times). Images were acquired using a Leica SP8 FALCON confocal microscope. Maximum intensity projection of Z-stack images was used for data presentation. All data were processed using ImageJ software.

### Live-cell imaging

20 × 10^4^ HeLa cells were seeded the day before treatment on a glass-bottom microwell dish (MatTek Corp.). HeLa cells were washed with 1 × PBS (2 times), then treated with **DBI** (500 nM) and the nuclear dye Hoechst 33342 (500 nM; Sigma-Aldrich, Ref. No.: B2261) dissolved in live cell imaging solution (Molecular Probes™, Ref. No.: A14291DJ) for 20 min at 37°C in 5% CO_2_ prior to imaging. Images were acquired using a Leica SP8 FALCON confocal microscope equipped with an incubation chamber operating at 37°C in 5% CO_2_. The images and data were presented and processed as described above.

### Digestion experiments

20 × 10^4^ HeLa cells were seeded the day before treatment on a glass-bottom microwell dish (MatTek Corp.). HeLa cells were treated with **DBI** (1 μM) or with an equivalent amount of DMSO (0.02% v/v) for 24 h and, when required, subjected to photo-irradiation using a LED cube (Ex: 470/22 nm) operating at 30 mW cm^−2^ for 20 min. Next, the cells were incubated for an additional 30 min at 37°C in 5% CO_2_ and fixed with 4% PFA, then permeabilized in 0.1% Triton X-100 at room temperature. Cells were treated with DNase I (Invitrogen, Ref. No.: EN0521) dissolved in 1 × PBS supplemented with 100 mM MgCl_2_ and incubated for 24 h at 37°C. Then, Hoechst 33342 (500 nM) was added to the cells for 1 h and fluorescence images were acquired using a Leica SP8 FALCON confocal microscope. The images and data were presented and processed as described above.

### DNA fiber analysis

Asynchronous HeLa cells were seeded at 25 × 10^4^ cells for 24 h. Then, cells were treated with **DBI** (1 μM) for 24 h. When required, **DBI**-treated HeLa cells were photo-irradiated using a LED cube (Ex: 470/22 nm) operating at 30 mW cm^−2^ for 5, 10 or 20 min. Then, the cells were incubated for an additional 30 min at 37°C in 5% CO_2_. After that, cells were pulse-labeled with IdU (25 μM), CIdU (200 μM), and thymidine (200 μM). Cells were then harvested and resuspended in cold PBS. DNA fiber stretching and subsequent immunostaining of DNA fibers were performed as previously described (45,54). DNA fibers were visualized using the Leica Thunder Widefield microscope, and images were captured randomly from different fields that contained untangled fibers. Only fibers containing IdU labels flanked by CIdU labels with intact ss-DNA ends were selected for analysis using LASX (Leica) and ImageJ software packages. Measurements were made in micrometers and converted to kilobases using a conversion factor for the length of a labeled track of 1 μm corresponding to roughly 2 kb.

### Immunofluorescence experiments

20 × 10^4^ HeLa cells were seeded the day before treatment on a glass-bottom microwell dish (MatTek Corp.). HeLa cells were treated with **DBI** (1 μM), Temoporfin (5 μM), or with an equivalent amount of DMSO (0.02% v/v) for 24 h and, when required, subjected to photo-irradiation using a LED cube (Ex: 470/22 nm) operating at 30 mW cm^−2^ for 20 min. Next, the cells were incubated for an additional 30 min at 37°C in 5% CO_2_ and fixed with 4% PFA, then permeabilized in 0.1% Triton X-100 at room temperature. Cells were exposed to blocking solution (1 × PBS and 2% nonfat milk) in a humidified chamber for 1 h at 37°C, and where indicated, incubated with anti-LAMP1 (Cell Signaling, Ref. No.: 9091, dilution 1:400), anti-EEA1 (Cell Signaling, Ref. No.: 3288, dilution 1:200), anti-CD63 (abcam, Ref. No.: ab8219, dilution 1:400), anti-8-oxoG (Sigma-Aldrich, Ref. No.: MAB3560, dilution 1:500), anti-BG4 (absolute antibody, Ref. No.: Ab00174-30.126, dilution 1:1000) or anti-γH2AX (Cell Signaling, Ref. No.: 9718, dilution 1:400) antibodies in blocking solutions (1% Tween 20, 1 × PBS and 5% goat serum (Sigma-Aldrich, Ref. No.: G9023)) for 2 h at 37°C. In the case of BG4 treatment, cells were further incubated with a rabbit antibody against the DYKDDDDK epitope (Cell Signaling, Ref. No.: 14793), at 1:800 dilution in a blocking solution (1% Tween 20, 1 × PBS and 5% goat serum) for 1 h at 37°C. Next, cells were incubated with goat anti-rabbit IgG (H + L) Alexa Fluor 594 (Life Technologies, Ref. No.: A11012) (dilution 1:1000) or goat anti-mouse IgG (H + L) Alexa Fluor 647 (Invitrogen, Ref. No.: A-21235) dissolved in blocking solution (1% Tween 20, 1 × PBS and 5% goat serum) for 1 h at 37°C. After each step, cells were washed three times for 5 min with 1 × PBS. For nuclear staining, cells were incubated with Hoechst 33342 (500 nM) for 1 h at room temperature. Cells were imaged using a Leica SP8 FALCON confocal microscope. Maximum intensity projection of Z-stack images was used for data presentation. All data were processed using ImageJ software.

### Dot blot assay for the detection of G4 structures

BG4 antibody was purified following a previously described protocol (60). DNA isolated from exosomes (5 ng/μl) or DNA oligonucleotides (6 ng/μl) were heated at 95°C in 100 mM KCl and allowed to cool overnight at room temperature. A total amount of 500 ng DNA was loaded onto a Hybond-N + membrane (GE Healthcare) using a Bio-Dot Microfiltration Apparatus (Bio-Rad). The membrane was blocked for 3 h at room temperature with 5% milk in intracellular salt solution (25 mM HEPES, pH 7.5, 10.5 mM NaCl, 110 mM KCl and 1 mM MgCl_2_) and incubated with BG4 antibody (1:1000 dilution) overnight at room temperature. After washing the membrane for 15 min twice with wash buffer (10 mM Tris, pH 7.4, 100 mM KCl, 0.1% (vol/vol) Tween 20), the membrane was incubated with an anti-FLAG antibody (Sigma-Aldrich, #F3165, 1:5000 dilution) for 2h and washed for 15 min twice with the wash buffer. The membrane was incubated with goat anti-mouse IgG (H + L) antibody, HRP (Thermo Fisher Scientific), and the dots were detected using a chemiluminescent reagent.

### Zebrafish maintenance

Zebrafish (Danio rerio) used in this study were maintained in compliance with the standard procedures at the Umeå University Zebrafish Facility. All animal experiments were approved by the Umeå animal experimental ethics board (Dnr A6-2020).

### Treatment of zebrafish embryos with DBI

Wildtype embryos were dechorionated and used either 12 h post-fertilization (hpf) or 24 hpf to test the cytotoxicity of **DBI***in vivo*. **DBI** was diluted in an E3 medium and an equal amount of DMSO was used as a control. Embryos at 12 hpf were transferred to 12 well-plates, treated with different concentrations of **DBI** (20 nM, 100 nM, 500 nM and 1 μM) and incubated in the absence of light for 12 h at 28.5°C. When required, the embryos were photo-irradiated by a blue-LED (55.6 mW cm^−2^) for 5 min. Then, the embryos were kept in the dark for 15 min and finally fixed in 4% PFA and stored overnight at 4°C. Embryos at 24 hpf were treated with **DBI** (10 μM) and incubated at 28.5°C in the absence of light for 24 h. When required, the embryos were photo-irradiated by a blue-LED (GFP filter, 55.6 mW/cm^2^) for 5 min. Then, the embryos were kept in the dark for 3 h and finally fixed in 4% PFA before storing overnight at 4°C. For the local activation experimental setup, embryos at 12 hpf were treated with 1 μM **DBI**, or DMSO (0.02% v/v) in E3 medium and incubated in the absence of light for 12 h at 28.5°C. Next, the live embryos were mounted in low-melt agarose and photo-irradiated with a Nikon A1 confocal microscope using a 20× water immersion lens and 20% of 488 nm laser activity in a specified local region covering 4 somites above the yolk sac extension, by 4.0 times zoom for 3 min, kept in dark for 15 min, and fixed in 4% PFA in PBS overnight at 4°C.

### TUNEL assay

Click-iT Plus TUNEL Assay for *in situ* apoptosis detection with Alexa Fluor 594 dye (Invitrogen, Ref. No.: C10618) was used following the manufacturer′s instructions. In short, fixed embryos were washed twice in PBS for 5 min followed by PBST (1% Tween 20) two times for 5 min. The embryos were then treated with proteinase K (10 mg/ml) (Thermo Fisher Scientific, Ref. No.: EO0491) for either 10 min (24 hpf embryos) or 30 min (48 hpf embryos) prior to fixation with 4% PFA for 20 min. Embryos were washed with PBST two times for 5 min and permeabilized for at least 1 h (three cycles of 20 min) with PBT (PBS, 1% Triton-X). Embryos were then incubated in terminal deoxynucleotidyl transferase (TdT) reaction buffer (Invitrogen, Ref. No.: C10618) for 10 min at RT. TdT reaction buffer was removed and replaced by TdT reaction mixture (TdT reaction buffer, EdUTP and TdT enzyme) and incubated for 2 h at 37°C. Embryos were washed with 3% BSA in PBS and incubated in a Click-iT plus TUNEL reaction cocktail (Invitrogen, Ref. No.: C10618) for 30 min at room temperature. Embryos were washed with 3% BSA in PBS before analysis.

### Immunofluorescence experiments in zebrafish embryos

Immunofluorescent staining was performed for both 12 hpf and 24 hpf embryos after the TUNEL assay. Embryos were washed using PBS Tween 20 (1% PBT) three times for 5 min. Embryos were pre-incubated in 0.75 ml blocking buffer (1% DMSO and 5% sheep serum in PBT) for at least 2 h at room temperature. The pre-blocking solution was removed and mouse anti-8-oxoG (Sigma-Aldrich, Ref. No.: MAB3560, dilution 1:200), rabbit anti-BG4 (absolute antibody, Ref. No.: Ab00174-30.126, dilution 1:200) and rabbit anti- FLAG M2 Antibody (Cell Signaling, Ref. No.: 14793, dilution 1:200), primary antibodies were added in 0.75 ml blocking buffer and incubated at 4°C overnight on a shaker incubator. Unbound primary antibody was washed using PBS Tween 20 (1% PBT) three times for 30 min and secondary antibodies, goat anti-rabbit Alexa Fluor 488 (1:500, Invitrogen Molecular probes), goat anti-mouse Alexa Fluor 488 (1:500, Invitrogen Molecular probes) and goat anti-mouse Alexa Fluor 633 (1:500, Invitrogen Molecular probes) were added in 0.75 ml blocking buffer and incubated at 4°C overnight on a shaker incubator. Unbound secondary antibody was washed using PBS Tween 20 (1% PBT) three times for 30 min and 4′,6-diamidino-2-phenylindole (DAPI) was used to visualize the nuclei. Stained embryos were kept in 70% glycerol. Images were taken using a Nikon confocal microscope.

### Exosome purification

Exosomes were purified from HeLa cells (1 × 10^6^) following the manufacturer's protocol of exosome purification using an Exo-spin™ mini kit (Cell guidance systems) (61). In brief, cells were grown into DMEM (Dulbecco's Modified Eagle Medium) media (Gibco) supplemented with 10% exosome-depleted FCS (Fetal calf serum) (Gibco) and penicillin-streptomycin solution (1×). Cells were first centrifuged at 300 × g for 10 min to collect the supernatant followed by another round of centrifugation at 16,000 × g for 30 min to remove any remaining cell debris. The supernatant was mixed with Exo-spin™ Buffer in a 2:1 ratio and incubated overnight at 4°C to increase the exosome yield. Then, the mixture was subjected to ultracentrifugation in a Beckman Coultier L-90K ultracentrifuge using SW41Ti Swinging bucket rotors at 20,000 × g for 1 h at 4°C and the pellet containing exosomes were resuspended into 50 μl of 1× PBS. The gravity spin-columns were first equilibrated with 1 × PBS at room temperature before loading with the exosome resuspension. The flow-through was discarded while the purified exosomes were eluted by adding 180 μl of PBS.

### Extraction of DNA from exosomes

To remove the DNA associated with the outer membrane of exosomes (25), we digested the external DNA by treating the purified exosomes with 0.15 units/μl DNase I (Invitrogen, Ref. No.: EN0521) for 30 min at 30°C. Then, we added 0.5 M EDTA and incubated at 70°C for 5 min to inactivate DNase I followed by 1× PBS wash at 100,000 × g for 70 min. The exosome-containing pellets were resuspended in 1× PBS. Then, we proceeded to the isolation of exosome DNA following a previously published protocol (62). In short, the exosomes were lysed in the presence of 0.5% SDS (sodium dodecyl sulfate), 50 mM Tris–HCl (pH 8.0), 0.1 M EDTA, 10 μg/ml RNase A (Thermo Fisher Scientific) and 0.1 mg/ml of proteinase K (Thermo Fisher Scientific) at 56°C for 3 h followed by phenol-chloroform extraction and ethanol precipitation. The isolated exosomal DNA was resuspended in 20 μl of ultrapure DNase-RNase-free water. The concentration and purity of exosomal DNA were analyzed using a NanoDrop™ One^C^ Microvolume UV-Vis Spectrophotometer and on 0.8% agarose gel.

### qPCR

To investigate the potential presence of guanine-rich DNA sequences forming G4 structures in the extracted exosomal DNA, we performed qPCR amplification using qPCRBIO SyGreen Mix Separate-ROC (PCR Biosystems, Ref. No.: PB20.14–05). PCR cycles were carried out with a 3 min denaturation step at 95°C followed by 35 amplification cycles of denaturation at 94°C for 15 s, annealing at 60°C for 20 s, and extension at 72°C for 30 s. Final PCR products were loaded and run on 1.5% agarose gels along with the 100 bp and 1 kb DNA ladders (GeneRuler, Thermo Fisher Scientific). *GAPDH* transcripts and *GAPDH* promoter levels were considered as controls while *GAPDH* promoter levels were used to normalize the variability in the levels of G4-rich sites in exosomal and genomic DNA. The PCR primers were designed in Primer3plus. Forward (F) and reverse (R) primer sequences of the different oncogene promoters and housekeeping genes are listed in Supplementary Table S4.

### Cryo-EM sample preparation and data collection

Purified exosomes in PBS buffer were vitrified on Quantifoil Cu R200 2/2 (Electron Microscopy Sciences, Ref. No.: Q2100CR2) grids. Prior to sample application, the grids were glow discharged using a Pelco easiGlow device (Ted Pella Inc.) at 15 mA for 30 s. Samples were deposited by transferring 3 μl of sample onto the glow-discharged side of the grid, blotted, then plunge frozen in liquid ethane, using a Vitrobot plunge freezer (Thermo Fisher Scientific), with the following settings: 22°C, 80% humidity, Blot-force = –5, 60 s wait time and a blotting time of 3 s. All data were collected on an FEI Titan Krios transmission electron microscope (Thermo Fisher Scientific) operated at 300 keV and equipped with a Gatan BioQuantum energy filter and a K2 direct electron detector. A condenser aperture of 70 μm and no objective aperture were chosen for data collection. Data were acquired in parallel illumination mode using EPU (Thermo Fisher Scientific) software at a nominal magnification of 33 kx (4.3 Å pixel size).

### Determination of the intracellular H_2_O_2_ level

5 × 10^3^ HeLa cells/well were seeded in complete medium on 96 well-plates 24 h before the treatment with **DBI**. HeLa cells were washed (2 times) with live cell imaging solution (Molecular Probes™, Ref. No.: A14291DJ), then treated with **DBI** (1 μM) or DMSO (0.02% v/v) dissolved in live cell imaging solution and incubated for 90 min at 37°C in 5% CO_2_. When required, the cells were irradiated with blue light using a LED light cube (Ex: 470/22 nm) operating at 30 mW cm^−2^ for 4 min. After irradiation, ROS-Glo™ H_2_O_2_ kit (Promega) was used according to the manufacturer's instructions and the luminescence was recorded on a Synergy H4 microplate reader (Biotek).

## RESULTS

### Photosensitizer design and characterization

**DBI** was prepared according to the one-pot synthetic route depicted in Figure 1B. Commercially available 2-methylnaphtho[1,2-d]thiazole was treated under basic conditions to generate the 1-aminonaphthalene-2-thiol that was then directly involved in a reaction with the 4-bromo-1,8-naphthalic anhydride. Next, isopentyl nitrite was added to the reaction mixture to afford ring-closure of the *π*-conjugated core *via* a Pschorr cyclization. Due to its poor solubility in common organic solvents, the resulting anhydride (**DBA**) was filtered off, dried, and directly utilized in the next step, *i.e*. the imidification in presence of pentan-3-amine. Once alkylated and purified (Supplementary Figures S1-3), single crystals of the isolated compound were successfully grown, confirming the structure of the target **DBI** (Figure 1C and Supplementary Figure S4 and Table S1). Owing to its fused and *π*-extended core, **DBI** shows an intense absorption band at 483 nm characterized by a molar absorptivity of *ca* 18 000 M^−1^ cm^−1^ and an emission band located in the green-yellow spectral region (*λ*_em_ = 544 nm) with a fluorescent quantum yield (*Φ*_F_) of *ca* 0.08 (Figure 1D and Supplementary Figure S5 and Table S2). **DBI**′s spectral signatures were found to be in good agreement with time-dependent density-functional theory (TD-DFT) computed spectra (Figure 1D). Low-temperature time-gated photoluminescence spectra recorded with a 50 μs delay revealed the presence of a triplet state, which manifests as a phosphorescence band centered at ∼700 nm (Figure 1D). Singlet oxygen generation measurements were thus subsequently carried out revealing a rather uncommon quantum yield close to unity (*Φ*_Δ_ = 0.95), for such a small heavy-atom-free molecule (Figure 1D and Supplementary Figure S6). Complementary theoretical investigation reveal that this efficient singlet-to-triplet intersystem crossing (ISC) mainly originates from the large spin-orbit coupling (SOC) computed between *S*_1_ and *T*_1_ (SOC of 7.7 cm^−1^, Figure 1E) itself activated by the distortion of the *π*-conjugated core induced by the lower phenyl ring (dihedral angle of *ca* 28°), releasing the selection rules (El Sayed's rules) that normally forbid the process in small organic molecules (63). Finally, despite the above-mentioned almost unitary singlet oxygen generation efficiency (which typically correlates with quenched luminescence), residual emission of **DBI** is enough to enable its potential detection inside cells and thereby its use as a ‘theranostic’ agent.

### DBI triggers intracellular ROS generation and photocytotoxicity

**Figure 2.**
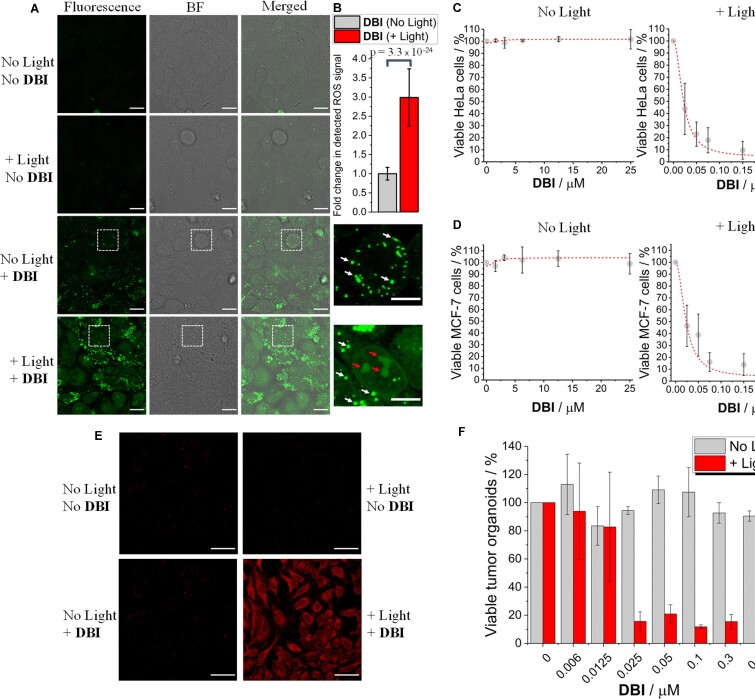
Intracellular ROS production by **DBI** and photo-driven cancer cell death. (**A**) HeLa cells were treated with **DBI** (1 μM) or with an equivalent amount of DMSO (0.02% v/v) and incubated at 37°C for 24 h. Where indicated, blue light generated by a LED light cube (30 mW cm^−2^) was applied to HeLa cells for 20 min. CellROX™ green reagent (5 μM) was added to the cells for 30 min at 37°C before PFA fixation. *λ_exc_*/*λ_em_*: 491/510–650 nm. Scale bar 20 μm. BF indicates bright field. The confocal images on the right are enlargements of the squares in the images on the left. Note that the extranuclear green granular fluorescent foci appearing in **DBI**-treated cells, indicated by the white arrows, are associated with **DBI** fluorescent signal. The nuclear/nucleolar green fluorescent signal, indicated by the red arrows, is due to **DBI** photo-induced oxidation of CellROX green reagent. Scale bar 10 μm. (**B**) Quantification of the nuclear ROS signal in non-irradiated and irradiated **DBI**-treated HeLa cells. Data represent populations of individual cells (*N* = 50 cells). Error bars indicate mean ± SD. Analysis of the data was performed using a two-sample *t* test and the *p* value is indicated. (**C, D**) Cytotoxic effects of **DBI** on HeLa or MCF-7 cells in the absence or presence of blue light (27 mW cm^−2^) irradiated for 6 min. Error bars indicate mean ± SD (*n* = 3). (**E**) LIVE/DEAD™ assay used to determine the viability of HeLa cells. HeLa cells were treated with **DBI** (1 μM) or with an equivalent amount of DMSO (0.02% v/v) and incubated at 37°C for 24 h. Where indicated, blue light was applied as in (C, D) and the cells were further incubated at 37°C for an additional 24 h. LIVE/DEAD™ fixable red stain for cytotoxicity detection (1 μl /ml) was added to the cells for 30 min at 37°C before PFA fixation. *λ*_exc_/*λ*_em_: 598/630–730 nm. Scale bar 50 μm. (**F**) Cytotoxic effects of **DBI** on murine pancreatic tumor organoids in the absence or presence of blue light (27 mW cm^−2^) irradiated for 6 min. Error bars indicate mean ± SE (*n* = 3).

Before investigating the photodynamic therapeutic efficacy of **DBI**, we evaluated its ability to generate cellular oxidative stress. CellROX™ green reagent is used as a fluorogenic probe for detecting oxidative stress in the nucleus of live cells (7). This dye is weakly fluorescent in a reduced state while displaying bright green fluorescence upon oxidation by ROS. As depicted in Figure 2A and B, human cervical epithelioid carcinoma (HeLa) cells treated with **DBI** (1 μM) and CellROX™ green reagent and irradiated with blue light, that also matches **DBI**′s absorption spectrum, showed a bright green fluorescent signal mainly localized in the cell nucleus supporting the ability of **DBI** to generate ROS. Importantly, control experiments with the CellROX™ green reagent performed in the absence of both **DBI** and light as well as in the presence of either **DBI** or light showed negligible ROS-associated nuclear fluorescent signal, indicating that intracellular ROS production only occurs in light-irradiated **DBI**-treated cells, thereby confirming its potential usefulness for the targeted applications.

Next, we determined the phototherapeutic efficacy of **DBI** toward HeLa and breast (MCF-7) cancer cell lines using the PrestoBlue™ cell viability assay (39,54). In the absence of light irradiation, no cytotoxicity was observed for either HeLa or MCF-7 cells treated with various concentrations of **DBI** (ranging from 0 to 25 μM) even after 48 h of continued drug exposure (Figure 2C, D). For photocytotoxicity studies, HeLa and MCF-7 cells were treated with various concentrations of **DBI** (ranging from 0 to 0.3 μM) for 24 h, irradiated for 6 minutes with blue light using a LED light cube (470/22 nm), and further incubated for an additional 24 h. These experimental conditions were optimized in order to avoid cell death caused by overexposure to light and to match the incubation time and general experimental conditions used in the dark cytotoxicity studies. We demonstrated that **DBI** exhibit high photocytotoxic activity with a half-maximum inhibitory concentration: IC_50_ (HeLa) = 22.5 ± 7.5 nM and IC_50_ (MCF-7) = 27.3 ± 11.0 nM and a high dark IC_50_/light IC_50_ phototoxic index (PI) ratio of ∼ 1000 (Figure 2C, D). The **DBI** photo-triggered cancer cell death was also confirmed using the LIVE/DEAD™ viability/cytotoxicity stain assay. As depicted in Figure 2E, only the **DBI**-treated HeLa cells subjected to light irradiation displayed a bright red signal indicative of cell death due to ruptured plasma membrane. Moreover, real-time monitoring of morphological changes in living HeLa cells subjected to **DBI** treatment and blue light irradiation unraveled dramatic alterations of the cellular architecture, characteristic of a very efficient PDT process (Supplementary Figure S7) (7).

The phototherapeutic activity of **DBI** was compared with 5,10,15,20-tetra(*m*-hydroxyphenyl)chlorin (mTHPC, Temoporfin) (64), the active pharmaceutical substance in the medicinal photosensitizer Foscan® used in the clinics for the treatment of head and neck cancer (64,65). By using the same experimental setting used for **DBI**, in the dark, Temoporfin-treated HeLa cells showed significant toxicity with an IC_50_ (HeLa) = 19.0 ± 1.3 μM (Supplementary Figure S8A). Light irradiation delivered by using a LED light cube operating at either 470/22 nm or 542/20 nm enhanced Temoporfin phototoxic activity with IC_50_ (HeLa, 470/22) = 0.43 ± 0.09 μM and IC_50_ (HeLa, 542/20) = 0.44 ± 0.11 μM for blue and green light, respectively (Supplementary Figure S8B, C). The PI index ratio was found to be < 50. Collectively, these findings suggest that **DB**I, in HeLa cells, possess enhanced phototherapeutic activity compared to Temoporfin. Finally, time-lapse experiments of live HeLa cells subjected to Temoporfin treatment and blue light irradiation clearly showed cell death associated with the PDT process (Supplementary Figure S8D).

The promising results obtained for **DBI** in 2D monolayer cell culture, prompted us to investigate the phototherapeutic efficiency of **DBI** in biologically relevant multicellular tumor organoids. It can be stressed that organoids represent a better structural arrangement of the cancer cells, where they can form cancer glands, with preserved basal/apical orientation, that better reflect the *in vivo* situation compared to the 2D monolayer cultures (58,66). Remarkably, **DBI** showed high phototoxicity towards murine pancreatic tumor 3D organoids with IC_50_ = 16.1 ± 7.5 nM that is close to the IC_50_ values obtained from the 2D monolayer human cell cultures (Figure 2F). These results confirm the high phototherapeutic efficacy of **DBI** even in complex media and, put alongside with its extremely low dark toxicity, and thus high phototoxic index of 1000, underline its potential relevance for therapeutic use.

### Live-cell imaging and immunofluorescence experiments reveal subcellular localization of DBI in ILVs

**Figure 3.**
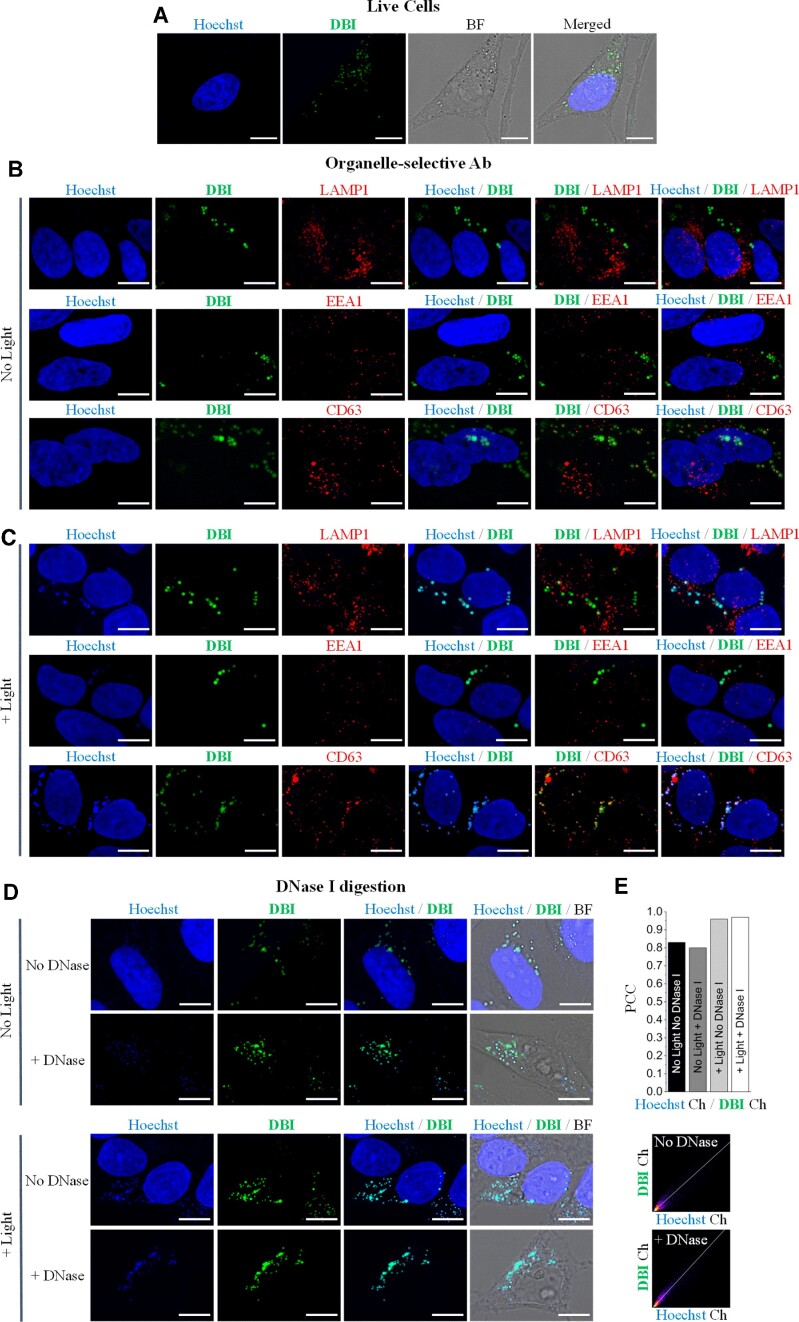
Cellular localization of **DBI** and response after photo-irradiation. (**A**) Live-cell imaging of HeLa cells treated with **DBI** (500 nM) and incubated for 20 min. HeLa cells were co-stained with the nuclear dye Hoechst 33342 (500 nM, blue signal). *λ*_exc_/*λ*_em_: 405/420–460 nm for Hoechst (blue signal); and 528/540–750 nm for **DBI** (green signal). The 2D single plane images were constructed by maximum intensity projection, where the highest intensity of each plane of Z-stacked images was used. The **DBI** signal that overlaps with Hoechst is not within the nucleus. Scale bar 10 μm. (**B, C**) Immunofluorescence experiments of HeLa cells treated with **DBI** (1 μM) for 24 h and either non-irradiated or irradiated with a blue LED light cube (30 mW cm^−2^) for 20 min and incubated for an additional 30 min at 37°C before PFA fixation. *λ_exc_*/*λ_em_*: 405/440–460 nm for Hoechst (blue signal); 528/540–590 nm for **DBI** (green signal); 598/620–750 nm for anti-LAMP1, anti-EEA1, and anti-CD63 (red signal). Scale bar 10 μm. (**D**) Confocal fluorescence images of HeLa cells treated with **DBI** (1 μM) for 24 h and either non-irradiated or irradiated with blue light using a LED light cube (30 mW cm^−2^) for 20 min and incubated for an additional 30 min at 37°C before PFA fixation. After cell fixation, DNase I was added to the cells that were incubated at 37°C for 24 h. Finally, Hoechst 33342 (500 nM) was used to detect DNA in cells. *λ*_exc_/*λ*_em_: 405/440–460 nm for Hoechst (blue signal); and 528/540–700 nm for **DBI** (green signal). Scale bar 10 μm. (**E**) Fluorescence co-localization analysis between Hoechst and **DBI** within ILVs. Upper panel: intensity histogram output of co-localization analysis between Hoechst and **DBI** quantified using the Pearson correlation coefficient (PCC). Lower panel: 2D scatter diagram for Hoechst and **DBI** channels under light-treated cell conditions.

The fluorescence images, acquired by confocal laser scanning microscopy (CLSM) in Figure 2A, indicated that **DBI** mainly localizes in the cytoplasm. Indeed, **DBI** was detected as bright foci inside the cytoplasm and absent within the nucleus of live cells (Figure 3A). To determine the exact subcellular-localization of **DBI**, we performed immunofluorescence experiments using a set of organelle-specific antibodies (Figure 3B, C). Lysosomal-associated membrane protein 1 (anti-LAMP1), early endosomal antigen 1 (anti-EEA1), and lysosomal-associated membrane protein 3 (anti-CD63) antibodies were used as lysosomes, early endosomes, and ILV markers, respectively. Hoechst was used to stain the DNA in the nucleus. Without light irradiation, no apparent co-localization between **DBI** and anti-LAMP1 or anti-EEA1 antibodies could be detected, excluding its accumulation in lysosomes and early endosomes (Figure 3B). Conversely, the **DBI** signal matched the anti-CD63 signal, suggesting that **DBI** localizes in the ILVs (Figure 3B and enlarged in Supplementary Figure S9).

### DBI and hoechst signal co-localize even after DNase-treatment

Next, we explored the intracellular distribution of **DBI** after light exposure. Under light-activated conditions, the signals of anti-CD63 and **DBI** remained overlapped (Figure 3C and enlarged in Supplementary Figure S9). Moreover, we observed that in both non-irradiated and even more in irradiated cells treated with **DBI**, the signal of the PS co-localized with the signal of Hoechst in the ILVs (Figure 3C–E; visible as turquoise color). Considering that Hoechst preferentially binds double-stranded DNA (*ds*-DNA), these findings suggest that **DBI** has the ability to target DNA molecules inside the ILVs.

To determine if **DBI** and Hoechst were bound on the surface or inside the ILVs, cells were treated with deoxyribonuclease (DNase) I (Figure 3D). In previous reports, DNase I has been shown to degrade the DNA inside the nucleus as well as the DNA that is attached to the outer membrane of the vesicles while keeping the intra-vesicular DNA content unchanged (25,31). In DNase I-treated cells, we observed that the fluorescence signal from the nucleus disappeared in both non-irradiated and irradiated conditions, indicating that the DNase I-treatment was successful (Figure 3D). However, the cytoplasmic signal from Hoechst and the **DBI** signal were unaffected, demonstrating that both **DBI** and Hoechst were localized inside the ILVs (Figure 3D and enlarged in Supplementary Figure S10). In addition, in light-treated cells, the fluorescent signal of Hoechst was enhanced compared to non-irradiated conditions, suggesting increased levels of DNA in the ILVs (Figure 3D, E). This is consistent with recent reports indicating that the use of genotoxic drugs increases the nuclear DNA abundance in exosomes (31). Collectively, our data provide a body of evidence of the uptake of **DBI** inside the ILVs and suggest that **DBI** causes DSBs and genomic instability through light-induced oxidative stress which results in increased amounts of DNA inside the ILVs, precursors of exosomes.

### G4s as potential recognition sites for DBI

**Figure 4.**
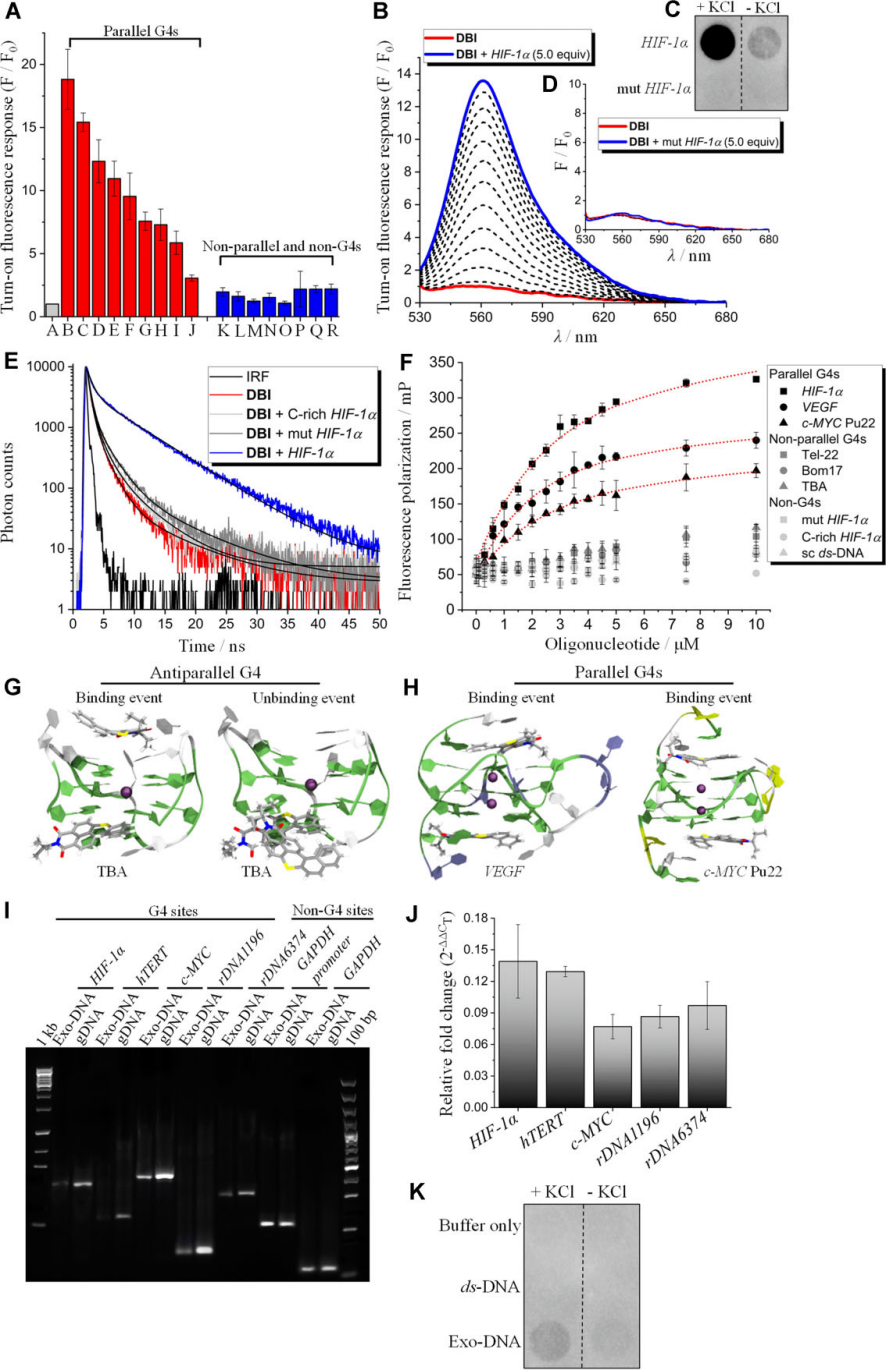
**DBI** binds to G4 structures. (**A**) **DBI**-associated turn-on fluorescence response upon individual addition of DNA structures. **DBI** (1 μM, DMSO 0.1% v/v) and oligonucleotides (5 μM) were mixed together in TRIS buffer (50 mM, pH 7.2) and KCl (100 mM) and allowed to equilibrate at 25°C for 30 min before collecting emission spectra. Signal normalization was performed by dividing the emission of the complex (F) over the emission of **DBI** alone (F_0_) at 561 nm. *λ*_exc_/*λ*_em_: 520/525–700 nm. **DBI** = A; *HIF-1α* = B; *c-MYC* Pu22 = C; *VEGF* = D; *HIF-1α* no flanking residues = E; *VAV-1* = F; *c-KIT* 2 = G; *CEB25* = H; *BCL*-2 = I; *c-MYC* Pu24T = J; Tel-22 = K; Bom17 = L; TBA = M; mut *HIF-1α* = N; C-rich *HIF-1α* = O; scr *ss*-DNA = P; *ds*-DNA *HIF-1α* no flanking residues = Q; *ds*-DNA *HIF-1α* = R. (**B**) Steady-state emission spectra of **DBI** (1 μM, DMSO 0.1% v/v) complexed with *HIF-1α* (from 0 to 5 μM) in TRIS buffer (50 mM, pH 7.2) and KCl (100 mM). (**C**) Dot blotting assay illustrating that a BG4 immuno-signal was clearly detected for *HIF-1α* but was almost undetectable in the mut *HIF-1α* control sequence. (**D**) Steady-state emission spectra of **DBI** (1 μM, DMSO 0.1% v/v) complexed with mut *HIF-1α* (from 0 to 5μM) in TRIS buffer (50 mM, pH 7.2) and KCl (100 mM). *λ*_exc_/*λ*_em_: 520/525–700 nm. (**E**) Time-resolved decay traces of **DBI** (0.5 μM, DMSO 0.01% v/v) complexed with *HIF-1α*, mut *HIF-1α* and C-rich *HIF-1α* (5 μM) in Tris buffer (50 mM, pH 7.2) and KCl (100 mM). *λ*_exc_: 490 nm. (**F**) Fluorescence polarization studies of **DBI** (1 μM, DMSO 1% v/v) in the presence of parallel, hybrid, and antiparallel G4s as well as non-G4 sequences (from 0 to 10 μM) in TRIS buffer (50 mM, pH 7.2) and KCl (100 mM). *λ*_exc_/*λ*_em_: 485 (±20)/620 (±40) nm. A superimposed dashed line in the **DBI**-*HIF-1α*,**DBI**-*VEGF* and **DBI**-*c-MYC* Pu22 systems is the result of nonlinear fitting with a 1:1 binding model. No quantitative data analysis was performed on **DBI** complexed with hybrid and antiparallel G4s as well as non-G4 sequences due to the absence of a well-defined optical response. (**G, H**) MD simulations for **DBI** complexed with antiparallel (thrombin binding aptamer (TBA), PDB: 1RDE (116)) and parallel (*VEGF*, PDB: 2M27 (117); *c-MYC*, PDB: 6O2L (118)) G4s. The G4 templates are represented in ribbons and the nucleobases are color coded as follows: guanine (green), thymine (white), cytosine (violet), and adenosine (yellow). **DBI** molecules are shown in stick representation and internal potassium cations are represented by a purple ball. In the anti-parallel G4 structure representing an unbinding event, unbound **DBI** interacts with the other **DBI** ligand. (**I**) Semi-quantitative PCR was used to amplify five G4-rich regions in the promoters of *HIF-1α*, *hTERT*, *c-MYC*, and in *45S* rDNA (rDNA1196 and rDNA6374) in the exosomes (Exo-DNA) and genomic (gDNA) DNA, purified from HeLa cells. A *GAPDH*-promoter and a *GAPDH* transcript having no G4 sites were used as internal controls. (**J**) Quantitative analyses (mean ± SD; *n* = 4) of the relative fold change in the levels of G4-rich sites in the promoters of *HIF-1α*, *hTERT*, and *c-MYC*, and in the *45S* rDNA in the exosomes compared to that of genomic DNA, normalized to the levels of *GAPDH*-promoter. (**K**) Dot blotting assay illustrating that the BG4 immuno-signal was clearly detected in isolated and purified exosomal DNA (500 ng) but almost undetectable in the *ds*-DNA control sequence.

Next, we examined **DBI**’s potential targets in the ILVs. Because **DBI** features a naphthalimide moiety and naphthalene diimide derivatives are a well-known class of G4-binders (67–69), we hypothesized that **DBI** may bind G4 DNA in the ILVs. The Raney lab has reported the presence of cytoplasmic G4 DNA induced by H_2_O_2_ that causes oxidative stress (70), suggesting that G4 DNA may also accumulate outside the nucleus. We therefore first studied the affinity of **DBI** for different G4 and non-G4 DNA substrates (G4s with various topologies, namely: parallel, hybrid and antiparallel, as well as single-stranded (*ss-*) DNA, and *ds*-DNA) (Supplementary Table S3). Fluorescence light-up sensing studies unraveled a binding preference of **DBI** for certain parallel G4 structures such as *c-MYC* Pu22, *VEGF*, and *HIF-1α* (Figure 4A). The partially distorted *π*-conjugated core of **DBI** and its uncharged state may account for the selectivity of **DBI** for a number of parallel G4s, which are known to have highly accessible G-quartet surfaces (39,45,71,72). The highest **DBI** turn-on fluorescent binding response was observed in the presence of the G4 motif found in the promoter of the hypoxia-inducible factor 1 alpha (*HIF-1α*) which forms a parallel G4 as demonstrated by circular dichroism (CD), ^1^H NMR spectroscopies, and dot blot assay using the BG4 antibody (Figure 4B, C and Supplementary Figures S11 and S12) (73). Fluorescence titration studies for **DBI** complexed with three control sequences; the first one bearing single point mutations (G-to-C) in each central G-tract that prevent G4 folding (mut *HIF-1α*), the second one being the complementary C-rich strand of *HIF-1α* (C-rich *HIF-1α*) and the third one being a self-complementary (sc) *ds*-DNA sequence, showed no significant **DBI** fluorescence enhancement indicating the discriminatory ability of **DBI** for certain parallel G4 structures (Figure 4A, C, D, and Supplementary Figures S13 and S14).

The ability of *HIF-1α* to enhance the emissive properties of **DBI** was further supported by time-correlated single photon counting (TCSPC) measurements (Figure 4E). Indeed, the intensity-weighted average lifetime (*τ_ave_*) associated with the decay traces of **DBI** (0.6 ns) upon the addition of *HIF-1α* increased to 3.0 ns, while it remained almost unchanged in the presence of C-rich *HIF-1α* (0.6 ns) or mut *HIF-1α* (0.7 ns), showing selectivity to *HIF-1α*. The increased fluorescence signal and longer lifetime of **DBI** bound to *HIF-1α* is likely due to a reduced conformational degree of freedom of **DBI** available in the excited state which opens to the formation of additional radiative pathways (74). This statement was supported by viscosity-dependent emission studies that showed that **DBI** fluorescence intensity is enhanced in high viscous media (Supplementary Figure S15) (45). In order to quantitatively determine the binding affinity (*K*_a_) of **DBI**, we performed fluorescence polarization (FP) studies using G4 DNA structures with various topologies as well as non-G4 DNA sequences (Figure 4F). Changes in molecular volume, resulting from the potential binding event between **DBI** and the DNA sequences, may reduce the extent of **DBI**′s molecular rotation providing high polarization values. **DBI** bound to *HIF-1α*, *VEGF*, or *c-MYC* Pu22 provided *K*_a_ = 4.6 × 10^5^ ± 0.5 M^−1^, 6.3 × 10^5^ ± 1.5 M^−1^ and 4.5 × 10^5^ ± 1.1 M^−1^, respectively. In all cases, curve fitting procedures based on a 1:1 stoichiometry model fits well to our experimental data. Consistent with fluorescence enhancement studies, no binding response was observed for **DBI** in the presence of non-parallel and non-G4 DNA structures. The calculated K_a_ for **DBI** bound to *HIF-1α*, *VEGF* or *c-MYC* Pu22 provides a high G4 over non-G4 discriminatory index when considering that no binding response could be detected for **DBI** complexed with the non-G4 control sequences (mut *HIF-1α*, C-rich *HIF-1α* and sc *ds*-DNA). However, it is important to note that the *K*_a_ values calculated for **DBI** complexed with *HIF-1α*, *VEGF*, or *c-MYC* Pu22 are similar to those reported for many G4-binding small-molecules (35,39,45,75,76) but are, nonetheless, lower when compared to those calculated for reference compounds and their analogues, such as PhenDC3 (77,78) and Pyridostatin (PDS) (78–80). PhenDC3 and PDS are cationic compounds and the introduction of positively charged moieties along the **DBI** scaffold may indeed enhance its binding strength toward G4 structures, a strategy that we are currently investigating in our laboratories.

We also investigated the potential binding of **DBI** to RNA sequences by FP studies (Supplementary Figure S16). In these experiments, three previously structurally characterized G4 RNA sequences (4G_3_U_3_, TERRA and *FMR1*) (81,82), and a control *ds-*RNA sequence (81) were used. No or very weak binding response could be detected for **DBI** complexed with RNA sequences both in double-stranded and G4 conformations, suggesting that **DBI** has low affinity to these RNA molecules.

To determine the potential ability of **DBI** to selectively stabilize G4s over duplex structures, we performed CD-based thermal assays (Supplementary Figure S17) (82,83). The thermal stability (Δ*T*_m_) of *VEGF* and *c-MYC* Pu22, in the presence of **DBI**, was increased ∼13 and 7°C, respectively. Conversely, under the same experimental conditions, **DBI** did not or weakly stabilized hybrid and antiparallel DNA G4 structures as well as RNA G4 and duplex sequences. Overall, these results are in good agreement with the fluorescence data and with the ability of **DBI** to preferentially bind to *VEGF* and *c-MYC* Pu22, and not to other DNA or RNA sequences.

### Molecular dynamics simulations predict the binding of DBI to G4s

Next, the binding modes of **DBI** with parallel and antiparallel G4s were investigated by molecular dynamics (MD) simulations (Figure 4G-H and Supplementary Table S5). Strikingly, we observed the occurrence of unbinding events in the **DBI**-anti-parallel G4 system in 3 over 10 replicas, while all **DBI** molecules remained bound on the parallel G4 structures, consistent with the experimentally observed selectivity (Figure 4G, H). Next, we determined the 1:1 molecular mechanism Poisson Boltzmann Surface Area (MM-PBSA) binding energy for each binding event (top- and bottom end-stacking mode), assuming that the presence of **DBI** in one binding site did not alter the conformation of the other binding site in either a cooperative or anti-cooperative way. Our results confirmed the **DBI** binding preference for parallel G4s. On average, we obtained MM-PBSA binding energies of –2.8 (5′-end) and –5.6 (3′-end) kcal·mol^−1^ for **DBI** complexed with the anti-parallel G4 and –7.6 (*VEGF*, 5′-end), –8.8 (*VEGF*, 3′-end), –10.5 (*c-MYC*, 5′-end) and –9.0 (*c-MYC*, 3′-end) kcal·mol^−1^ for **DBI** bound to parallel G4 structures. These data clearly indicate that the **DBI** binding energy for the anti-parallel G4 is dramatically lower compared to those obtained for parallel G4 conformations. These data correlate well with the experimental findings and support an end-stacking interaction mode between **DBI** and parallel G4s (Supplementary Figure S18). The end-stacking binding mode is demonstrated for other G4–ligand complexes, such as hemin-G4 or PhenDC3-G4 complexes (84,85). In addition, the Heme-G4 complex also showed catalytic activity involving oxygen atom transfer from H_2_O_2_ to a variety of substrates (86). Hemin can be displaced by PhenDC3 from the G4 DNA, supporting the structural models of both hemin and PhenDC3 to be stacking with the terminal G-tetrads (85). Therefore, to further validate if **DBI** interacts with the terminal end of the G4, we performed fluorescent displacement assay with the **DBI**-*HIF-1α* complex and PhenDC3 (Supplementary Figure S19). Indeed, the gradual addition of PhenDC3 into the binary **DBI**-*HIF-1α* complex reduced the fluorescence signal from the **DBI**-*HIF-1α* complex, demonstrating that PhenDC3 displaced **DBI** from the G4 template. These data show that the two small molecules, **DBI** and PhenDC3, compete for the same binding site, supporting the end-stacking mode found by our MD simulations.

### G4 structures are present in purified exosomal DNA

The accumulation of **DBI** in DNA-rich ILV sites along with its ability to bind parallel G4 structures with high selectivity prompted us to investigate the potential presence of G4-rich sequences in ILVs that are released into the extracellular space, the exosomes. Therefore, we isolated exosomes from HeLa cells, confirmed their integrity by cryo-electron microscopy (cryo-EM), then extracted and purified the exosomal DNA (Supplementary Figure S20). We used quantitative polymerase chain reactions (qPCR) to amplify the G-rich regions of different oncogene promoters (*c-MYC*, *hTERT* and *HIF-1α*) and ribosomal DNA (*45S* rDNA) that are reported to harbor G4 structures (Figure 4I,J and Supplementary Table S4). Considering that DNA in exosomes reflects the genomic and mutational status of parent tumor cells (25), we also amplified the corresponding G4-forming regions at genomic DNA to determine the occurrence of G4-rich sites in the exosomes. We observed the existence of G4-rich sequences in exosomal DNA which had similar sizes to the corresponding regions of genomic DNA, although their relative levels, as expected, were significantly lower compared to those found in the genomic fraction. Further evidence directed to investigate the potential presence of G4-forming sequences in exosomal DNA was provided by dot blotting assays using the BG4 antibody in the presence or absence of KCl (Figure 4K). A clear dot signal was obtained from the isolated and purified exosomal DNA, which was enhanced in the presence of KCl, as monovalent ions, such as K^+^, ensure G4 stabilization (Figure 4K). Overall, these data indicate the presence of G4s in the exosomes and suggest that **DBI** targets G4 DNA structures that are present in the vesicles that give rise to exosomes.

### Photo-activation of DBI reduces replication fork speed in response to DNA damage and increased G4 formation

**Figure 5.**
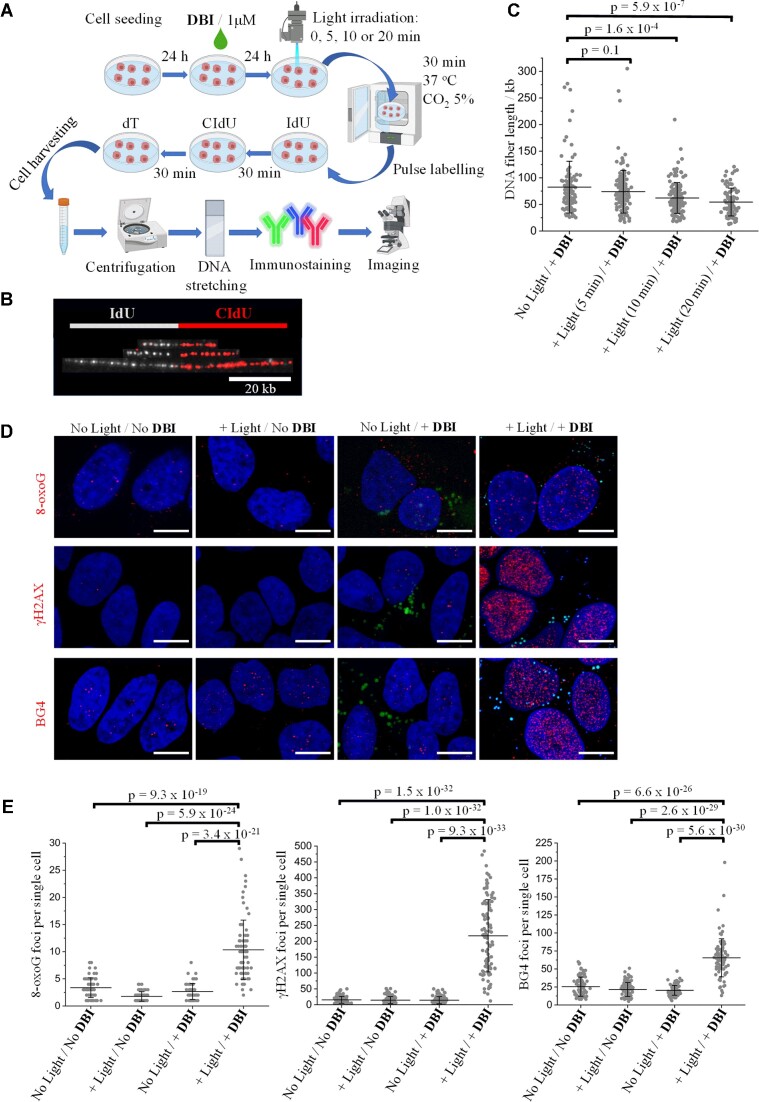
**DBI** photo-induces replication blockage and DNA damage through guanine oxidation and G4 formation. (**A**) Schematic illustration of the DNA fiber analysis experiment. Created by Biorender.com. (**B**) Representative images of replication tracts with different lengths. DNA fibers displaying 5-iodo-2′-deoxyuridine (IdU, white color) flanked by 5-chloro-2′-deoxyuridine (CIdU, red color). (**C**) Quantification of the DNA fiber length (μm) in **DBI**-treated HeLa cells kept in the dark or photo-irradiated with a blue LED light cube (30 mW cm^−2^) at different times (*t* = 5, 10 or 20 min). Data represent populations of individual DNA fibers (*t*_No light_ = 107, *t*_5min_= 148, *t*_10min_= 140 and *t*_20min_= 97). Error bars indicate mean ± SD. Analysis of the data was performed using a two-sample *t* test and the *p* value is indicated. (**D**) Immunodetection of G oxidation, DNA damage, and G4 formation in HeLa cells treated either with **DBI** (1 μM) or DMSO (0.02% v/v) for 24 h. The cells were irradiated with blue light using a LED light cube (30 mW cm^−2^) for 20 min and incubated for an additional 30 min at 37°C before PFA-fixation. The control cells were not irradiated but otherwise treated as described above. HeLa cells were co-stained with the nuclear dye Hoechst 33342 (500 nM, blue signal). *λ*_exc_/*λ*_em_: 405/440–460 nm for Hoechst (blue signal); 528/540–590 nm for **DBI** (green signal); 598/620–750 for BG4 and γH2AX (red signal); and 653/660–750 nm for 8-oxoG (red signal). Scale bar 10 μm. (**E**) Quantification of 8-oxoG, γH2AX and BG4 nuclear foci in the experimental conditions provided in (D). Data represent populations of individual cells (*N* = from 69 to 122 cells per condition). Error bars indicate mean ± SD. Analysis of the data was performed using a two-sample *t* test and the *p* value is indicated.

To gain insight into the phototherapeutic activity of **DBI**, we performed DNA fiber analysis (Figure 5A, B). This assay allows the replication fork progression to be monitored in individual DNA molecules (Figure 5B). Cells treated with **DBI** (1 μM) in the absence of light showed an average DNA fiber length of 82.5 μm (Figure 5C), indicating normal cell growth (45,54). In contrast, cells treated with **DBI** (1 μM) and photo-irradiated with blue light for 5, 10, or 20 min, displayed shorter mean lengths *t*_5min_ = 73.9 kb (*p* = 0.1), *t*_10min_ = 62.1 kb (*p* = 1.6 × 10^−4^) and *t*_20min_ = 54.5 kb (*p* = 5.9 × 10^−7^), indicating that **DBI** reduces fork speed in a light-dependent manner (Figure 5C).

The electron-rich character of guanine with its associated lowest reduction potential among all the nucleobases provide easy access of guanines to form 8-oxoGs upon oxidative stress. Thus, by performing CLSM, we set out to establish whether **DBI**′s photo-induced cancer cell death and slower replication fork speed are linked to its ability to induce DNA damage through ROS production. Using the same conditions as in the DNA fiber analysis, we first performed CLSM with an anti-8-oxoG-selective antibody to determine the ability of **DBI** to induce 8-oxoG. Photo-irradiated **DBI**-treated HeLa cells showed significant enrichment in 8-oxoG formation (∼3.1-fold) compared to mock-treated cells (*p* = 9.3 × 10^−19^), indicating increased levels of oxidative damage to the genome (Figure 5D, E). Next, we determined if photo-activation of **DBI** also induces DSBs. We used an antibody that recognizes phosphorylated histone H2AX (γH2AX) variants, an established marker that monitors DSBs (Figure 5D, E). Using CLSM, we observed ∼11.3-fold increased levels of γH2AX foci in photo-irradiated **DBI**-treated cells compared to the control cells (*p* = 1.5 × 10^−32^) already 30 min after light-activation of **DBI**, showing that photo-activated **DBI** not only induces oxidative DNA base damages, but also DSBs. Next, we determined by CLSM if Temoporfin also induces DNA breaks by performing similar experiments as for **DBI**. No significant variation in γH2AX foci could be detected in photo-irradiated Temoporfin-treated cells compared to the control cells (Supplementary Figure S8E, F) (65,87). In fact, even cells highly damaged by the treatment of Temoporfin and light did not provide changes in γH2AX signal (Supplementary Figure S8G). These data indicate that different cellular responses may be involved with the treatment of **DBI** or Temoporfin, the former being capable of generating DNA damage upon light illumination. Therefore, the induction of DNA damage seems not to be a general property of all PSs, and needs to be tested separately for each PS.

Many cancer-associated genes are regulated by G-rich sequences that are capable, under oxidative stress conditions, of refolding from a canonical duplex into G4 structures (51). This process, studied thoroughly in recent literature by Burrows′ group, involves a fifth G-track that acts as a ‘spare-tire’, facilitating extrusion of a damaged G-run (88). In addition, G4 foci are enriched in the genome when the latter is more prone to be single-stranded, i.e. during DNA replication (42), transcription (46), and DNA repair when resection takes place by EXO1 exonuclease (89). Therefore, we hypothesized that the combination of increased 8-oxoG levels as well as the increased levels of ssDNA presence during the resection of DSB repair, would induce increased G4 formation in the nucleus. We, therefore, investigated the potential interplay between guanine oxidation, DSBs and G4 formation. In fact, by using the G4-specific antibody BG4 (42), we were able to detect about 2.6-fold higher levels of nuclear BG4 foci in **DBI**-treated HeLa cells subjected to light irradiation compared to the controls (*p* = 6.6 × 10^−26^) (Figure 5D, E), demonstrating the induction of G4 levels.

As G4s are known obstacles to DNA replication progression and can stall DNA polymerases and thereby induce DNA damage in the form of SSBs or DSBs, the increased levels of G4 structures in the genome may also be interpreted as a trigger for DSBs, if the levels of G4s are too high for specialized G4 helicases to resolve these structures in a timely manner (53,90–96). In fact, as the levels of DSBs are significantly higher than the 8-oxoG levels in light-activated **DBI**-treated cells, the increased levels of G4 formation may also have triggered DSBs. Overall, these data suggest that **DBI**-photogenerated ROS are prone to result in mutagenic events, in which the oxidation of guanine bases in G-rich genomic regions can induce DNA-damage activation and G4 formation. A schematic representation of the potential **DBI**-photoinduced DNA damage pathways is illustrated in Supplementary Figure S21.

### *In vivo* validation of DBI photo-induced cytotoxic effect in zebrafish embryos

**Figure 6.**
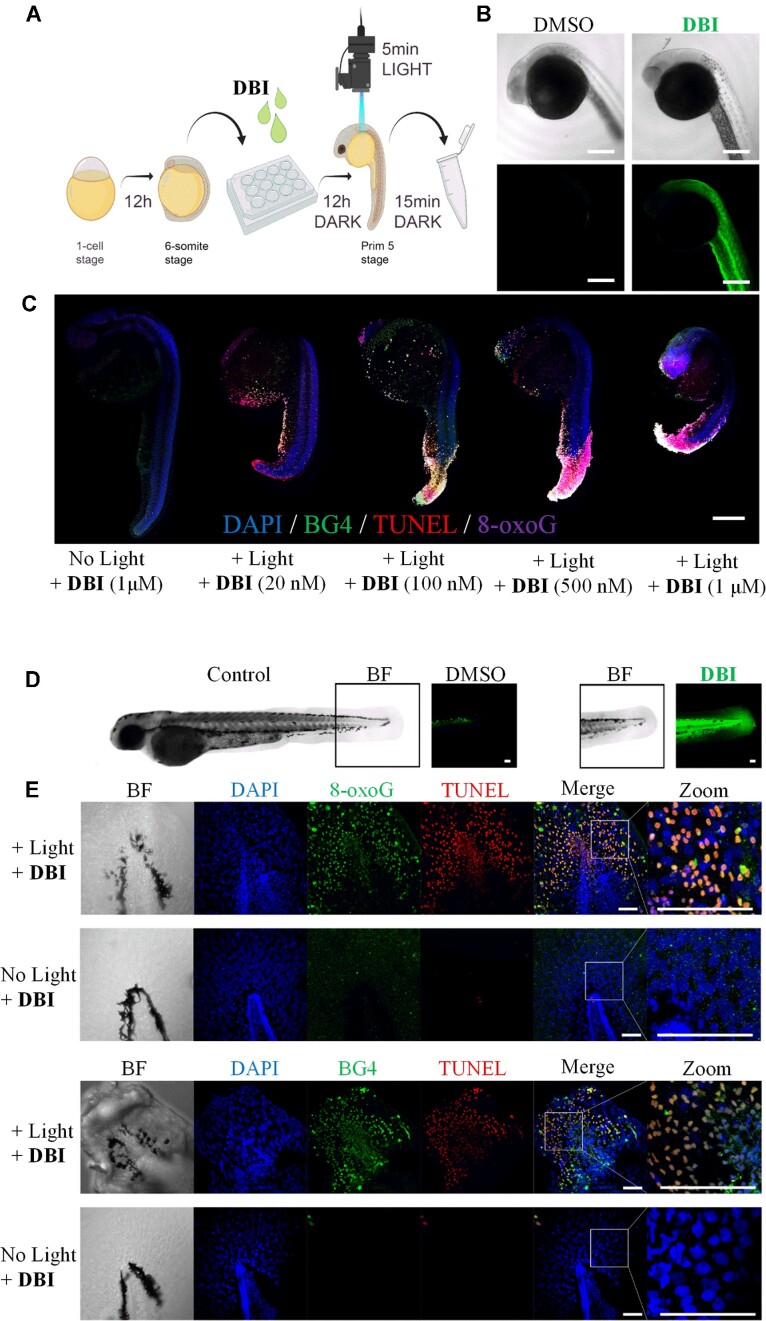
**DBI** photo-induced cell death in zebrafish embryos. (**A**) Schematic illustration of the treatment of zebrafish embryos. Created by Biorender.com. (**B**) BF (left) and CLSM (right) images of mock- and **DBI**-treated embryos. (**C**) Photo-induced toxicity of zebrafish embryos incubated with different concentrations of **DBI**: 20 nM, 100 nM, 500 nM and 1 μM. A blue LED operating at 55.6 mW cm^−2^ for 5 min was used to irradiate the embryos and induce DNA damage and cell death. Co-localized signals among DAPI (blue signal), BG4 (green signal), TUNEL (red signal) and 8-oxoG (magenta signal) are shown. Scale bar: 150 μm. (**D**) BF and CLSM images of zebrafish embryos, treated with 10 μM **DBI** from 24 hpf to 48 hpf showed **DBI** (green) accumulation in the tail. (**E**) BF and CLSM images showing the tails of the embryos treated with **DBI** (10 μM) in the dark or upon blue light exposure (55.6 mW cm^−2^ for 5 min). Single and co-localized channels are shown. DAPI (blue signal), 8-oxoG (green signal), BG4 (green signal) and TUNEL (red signal). Scale bar: 50μm.

Zebrafish are relevant models for human drug discovery not only because of the conserved physiology between humans and zebrafish (97), but also because about 70% of the human protein-coding genes have a zebrafish gene orthologue (98), and drug metabolism pathways are conserved (97). Zebrafish embryos are also translucent and allow real-time monitoring of drug uptake. Taking advantage of **DBI** characteristic emission, we first monitored its accumulation in zebrafish. Wildtype zebrafish embryos were dechorionated at the 6-somite stage, twelve hours post fertilization (hpf), and treated with **DBI** in the embryo medium for twelve hours in the dark until they reached the prim-5 stage (24 hpf) (Figure 6A). The **DBI**-treated 24 hpf embryos showed a widespread fluorescence signal along the whole embryonic body confirming effective uptake of the drug (Figure 6B). We next investigated the photocytotoxic effect of **DBI** on the embryos treated with different concentrations of **DBI** after light irradiation. Already after 15 min post light treatment, morphological changes were detected in the embryos, particularly in the tails, with an increasing severity correlated to increased **DBI** concentration (Figure 6C). Importantly, these morphological changes were not detected in the no light-treated embryos even at the highest **DBI** concentration treatment (Figure 6C). These data demonstrate an efficient phototoxicity of **DBI** *in vivo*, and suggest a similar light-induced effect of **DBI** in a living animal as found in our cell culture-based experiments.

To gain insights into the potential conservation between the observed effects detected in human cell cultures and zebrafish, we performed immunofluorescence experiments with the BG4 and 8-oxoG antibodies, and terminal deoxynucleotidyl transferase (TdT) dUTP Nick-End Labeling (TUNEL) assays to detect apoptotic DNA fragmentation (Figure 6C). **DBI**-treated embryos subjected to light exposure clearly showed an increased level of fluorescence signals associated with TUNEL, BG4, and 8-oxoG compared to mock-treated embryos, again highlighting the ability of **DBI** to induce cytotoxic effects in an exclusively light-dependent manner (Figure 6C). Damage sites and dying cells were detected throughout the embryos, with an emphasis on the tails and the most superficial tissue layers (Figure 6C). This last finding may be correlated with the high proliferative activity of the tails of the embryos at this particular growth stage.

### PDT effect of DBI is restricted to the compound-targeted area

As a proof-of-concept for the use of **DBI** at the targeted tissue area, which is the ultimate goal in PDT, a similar set of experiments were performed using 24 or 48 hpf embryos. Local photo-activation of **DBI** in 24 hpf embryos was achieved by irradiating the live embryos only within the specified region of interest (Supplementary Figure S22). Only the embryos treated with **DBI** and subjected to light irradiation showed an increased level of fluorescence signal associated with TUNEL. The TUNEL signal was only elevated in the light irradiated area, highlighting the possibility to trigger the PDT action only at a restricted site. In 48 hpf embryos, **DBI**-associated fluorescence signal is mostly confined within the tails with almost no accumulation in other compartments (Figure 6D). Based on our results from the 24 hpf embryos, we speculated that only this tail region with **DBI** accumulation may be affected after light exposure. Indeed, these photo-irradiated **DBI**-treated embryos showed DNA damage primarily confined in the tails indicating that the photo-driven toxic effects exerted by **DBI** could be again restricted only to the compound-targeted area (Figure 6E and Supplementary Figure S23). Importantly, embryos treated with **DBI** in the dark (no light exposure) neither showed significant morphological alterations nor did they provide any indication of DNA damage (Figure 6E). Together, these data suggest **DBI** as a promising PS with minimal side-effects into untreated areas and highlight the benefits of **DBI** in PDT.

### Increased levels of H_2_O_2_ in photo-irradiated cells

To determine if diffusible ROS, such as H_2_O_2_, may be produced in cells upon **DBI** photoactivation, and that elevated levels of H_2_O_2_ could be one of the reasons behind **DBI**’s phototoxicity, we performed a bioluminescence assay that measures the intracellular level of H_2_O_2_ (Supplementary Figure S24) (3). Indeed, the H_2_O_2_ level in photo-irradiated **DBI**-treated cells increased ∼ 12-fold compared to mock-treated cells, demonstrating the ability of **DBI** to photogenerate H_2_O_2_ (3). This suggests that a possible electron transfer mechanism towards molecular oxygen, resulting in the formation of superoxide O_2_^•−^, a precursor that turns into H_2_O_2_, via for instance superoxide dismutase, could take place in addition to the energy transfer that leads to the production of singlet oxygen (8). Both processes would then play an important role in photoinduced cell death. Finally, a confirmation of this dual mechanism of ROS formation, that would proceed upon both ^1^O_2_ and superoxide O_2_^•−^, was provided by electron paramagnetic resonance (EPR) *in vitro* studies. EPR, in the presence of scavengers or ‘spin-traps’, enables to detect short lived radical or intermediates such as O_2_^•−^ or ^1^O_2_. 2,2,6,6-tetramethyl-4-piperidine (TEMP) is a classically used scavenger that reacts with ^1^O_2_ and leads to the formation of TEMPO, a stable radical with a typical signature (99). As expected, this signature was observed upon *in-situ* irradiation of **DBI** in the presence of TEMP, and showed rapid growth upon prolonged irradiation time (Supplementary Figure S25A). When similar experiments were conducted using 5,5-dimethyl-1-pyrroline N-oxide (DMPO), intense O_2_^•−^ formation was detected as its characteristic adduct with DMPO (Supplementary Figure S25B). These results were confirmed by simulations which showed a single radical adduct species which g factor and hyperfine coupling that was identical to reported values for DMPO adducts of O_2_^•−^ (Supplementary Figure S25C; g = 2.006, a_N_ = 12.85 G, a_H_ = 10.35 G, a_H_ = 1.28 G) (100). Finally, EPR experiments performed in the absence of spin traps showed traces of a **DBI**-centered radical, similar in nature to what we recently reported for closely related molecules (Supplementary Figure S25D) (7).

## DISCUSSION

PDT is a clinically promising phototherapeutic modality for cancer treatment owing to its minimal invasiveness and low systemic toxicity in the absence of light (1,2). In recent years, a number of organelle-selective PSs targeting the nuclei, mitochondria, and lysosomes have been reported and their phototherapeutic properties have been demonstrated (11). Indeed, compared to non-selective PSs, the use of organelle-selective PSs provides a more efficient method to kill cancer cells.(11) However, organelle-targeted PSs with photoinduced cancer ablation capacity at concentrations in the nanomolar range are still very rare and usually incorporate toxic heavy atoms to enhance intersystem crossing, raising concerns about costs and safety (11). In cancer biology, much research has focused on the nucleus, considered the hearth of the cell because its connection with essential functions including storage and organization of the genetic material, DNA synthesis, transcription, and RNA processing (101). Yet, nucleus-targeted PDT agents are still rare (11), difficult to synthesize (102), have shown considerable dark cytotoxicity (14), and tendencies to cause genetic variations (13), limiting their applications. Nevertheless, other methods that involve localized oxidative nuclear damage are developed (103–105). For instance, recently the Lan laboratory designed a chimeric protein where the photosensitive KillerRed (KR) chromophore is fused with the telomere repeat binding factor 1 (TRF1), and demonstrated that this chimeric protein is capable of generating ROS upon visible light irradiation specifically at the telomeres (103). These data showed that the light-induced telomeric DNA damage results in severe consequences for cellular proliferation and survival, especially in telomerease-negative cell lines. Even more recently, the Opresko lab developed a precise chemoptogenetic tool that uses fluorogen-activating peptides (FAPs) with high affinity for the photosensitizer di-iodinated malachite green (MG2I) (104,105). They showed that upon FAP binding, MG2I photogenerates ^1^O_2_ at telomeres without causing damage elsewhere in the genome, and this results in replication stress and genome instability. These studies demonstrate how oxidative stress may drive genome instability and premature senescence, and support the importance of the development of new therapeutics that induce ROS to efficiently kill cancer cells. Here, in our study, we have designed a heavy-atom-free PS (**DBI**), which, by targeting two cancer-specific mediators/markers (DNA-rich tumor-derived ILVs/exosomes (25) and DNA G4 structures (106)), has the potential to maintain the similar mechanism of photodamage shown for the above described telomere-targeting studies and also for other nuclear DNA-damaging agents (i.e. DNA replication stress associated with increased levels of DNA damage). Consistent with recent reports that showed that genotoxic drugs increase the nuclear DNA abundance in exosomes (31), we found a higher fluorescent signal from Hoechst in photo-induced **DBI**-treated cells compared to non-irradiated conditions, suggesting elevated levels of DNA in the ILVs. At this point, we have not studied the mechanisms behind how these DNA fragments are cleaved and transported to the ILVs, however recent work from the Karin laboratory (107), showed that oxidized mitochondrial DNA fragments that exit mitochondria need to be cleaved by the flap-structure-specific endonuclease (FEN1). From this perspective, examining FEN1’s role in cleaving the damaged DNA fragments in the nucleus would be interesting for future studies.

By considering that the amount of DNA content into tumor-derived exosomes far exceed that found in normal cells (25,31) and, put alongside with our evidences that the extracted exosomal DNA contains potential G4 motifs, it underlines the potential relevance of targeting these vesicles for oncology ablation modalities. In addition, guanine has the lowest redox potential among the four DNA bases and runs of guanines render these sequences even more prone to oxidation (51,108) providing opportunities to maximize DNA damage by using G4-specific PSs.

The molecular pathways underlying how **DBI**′s phototoxicity develops inside the ILVs and is transferred to the nucleus require further investigations, however, it seems to involve concerted effects from singlet oxygen and other photogenerated ROS such as H_2_O_2_ (8,109). In fact, H_2_O_2_ has a long half-life and can diffuse into the nucleus causing oxidative damage to DNA that, if left unrepaired, may in turn stop DNA replication (51,70). Indeed, we provided evidence of elevated levels of H_2_O_2_ in photo-irradiated **DBI**-treated cells compared to mock-treated cells, which was also confirmed *in vitro* by EPR studies.

To conclude, we believe that **DBI** with its controlled accumulation in ILVs, selective binding to G4 structures, induction of G4 formation, guanine oxidation and DSBs, and enhanced photocytotoxicity shows great potential as a novel photoactivated anticancer agent. These properties may pave the way toward more personalized and efficient photodynamic cancer therapy procedures that focus on patients with mutations in genes encoding specialized G4 helicases (110), such as the FANCJ (111) and PIF1 (112,113) helicases, and defects in DDR machinery (114,115).

## DATA AVAILABILITY

The datasets generated during and/or analysed during the current study are available from the corresponding authors on reasonable request. The structure is deposited in CCDC and structure identifier number is 2083069.

## Supplementary Material

gkad365_Supplemental_File
