# Supplementary material for: A new G-quadruplex-specific photosensitizer inducing genome instability in cancer cells by triggering oxidative DNA damage and impeding replication fork progression
Source: Nucleic Acids Res. 2023 May 16;51(12):6264–85. doi: 10.1093/nar/gkad365 (PMC10325911; doi:10.1093/nar/gkad365)
Supplement: gkad365_Supplemental_File [file gkad365_supplemental_file.pdf]

Supporting information

A new G-quadruplex-specific photosensitizer inducing genome instability in cancer cells by triggering oxidative DNA damage and impeding replication fork progression

Marco Deiana,*[1] José María Andrès Castán,[2] Pierre Josse,[2] Abraha Kahsay,[3] Darío Puchán Sánchez,[2] Korentin Morice,[2] Natacha Gillet,[4] Ranjitha Ravindranath,[4,5] Ankit Kumar Patel,[6,7] Pallabi Sengupta,[1] Ikenna Obi,[1] Eva Rodriguez Marquez,[3] Lhoussain Khrouz,[4] Elise Dumont,[4,8] Laura Abad Galán,[4] Magali Allain,[2] Bright Walker,[9] Hyun S. Ahn,[10] Olivier Maury,[4] Philippe Blanchard,[2] Tangui le Bahers,[4] Daniel Öhlund,[6,7] Jonas von Hofsten,[3] Cyrille Monnereau,*[4] Clément Cabanetos,*[2,11] and Nasim Sabouri*[1]

[1] *Department of Medical Biochemistry and Biophysics, Umeå University, SE-901 87 Umeå, Sweden*
[2] *Univ Angers, CNRS, MOLTECH-ANJOU, SFR MATRIX, F-49000 Angers, France*
[3] *Department of Integrative Medical Biology, Umeå University, SE-901 87 Umeå, Sweden*
[4] *ENS de Lyon, CNRS, Université Claude Bernard Lyon 1, Laboratoire de Chimie UMR 5182, F-69342 Lyon, France*
[5] *Indian Institute for Science Education and Research (IISER), Tirupati-517507, India*
[6] *Department of Radiation Sciences/Oncology, Umeå University, SE-901 87, Umeå, Sweden*
[7] *Wallenberg Centre for Molecular Medicine (WCMM), Umeå University, SE-901 87, Umeå, Sweden*
[8] *Institut Universitaire de France, 5 rue Descartes, 75005 Paris, France*
[9] *Department of Chemistry, Kyung Hee University, Seoul, 02447, Republic of Korea*
[10] *Yonsei University, 50 Yonsei-ro, Seodaemun-gu, Seoul, Republic of Korea*
[11] *Building Blocks for FUture Electronics Laboratory (2BFUEL), IRL2002, CNRS -Yonsei University, Seoul, South Korea*
* corresponding authors
Email: marco.deiana@umu.se; cyrille.monnereau@ens-lyon.fr; clement.cabanetos@univ-angers.fr; nasim.sabouri@umu.se

General methods

Reagents and chemicals from commercial sources were used without further purification. Solvents were dried and purified using standard techniques. Silica gel chromatographies were performed on Aldrich (Saint-Louis, USA) silica gel (technical grade, pore size 60 Å, 230-400 mesh particle size) packed with analytical-grade solvents. Flexible plates ALUGRAM® Xtra SIL G UV254 from MACHEREY-NAGEL (Düren, Germany) were used for TLC. Compounds were detected by UV irradiation (Thermo Fisher Scientific, Waltham, MA, USA). NMR spectra were recorded with an AVANCE III 300 (1H, 300 MHz and 13C, 75MHz) from Bruker (Bruker, Billerica, MA, USA). Chemical shifts are given in ppm relative to TMS and coupling constants J in Hz. UV-vis spectra were recorded on a Shimadzu UV-1800 spectrometer (Shimadzu, Kyoto, Japan). High-resolution mass spectrometry (HRMS) was performed with a JEOL JMS-700 B/E (JEOL, Peabody, USA). Cyclic voltammetry was performed using a Biologic SP-150 potentiostat with positive feedback compensation in 0.10 M Bu_4NPF_6/CH_2Cl_2 (HPLC grade). Experiments were carried out in a one-compartment cell equipped with a platinum working electrode (2 mm of diameter) and a platinum wire counter electrode. A silver wire immersed in 0.10 M Bu_4NPF_6/CH_2Cl_2 was used as pseudo-reference electrode and checked against the ferrocene/ferrocenium couple (Fc/Fc+) before and after each experiment. The potentials were then expressed vs Fc/Fc+.

Synthesis and characterization of DBI

Synthesis of 2-(pentan-3-yl)-1*H*-benzo[7,8]thioxantheno[2,1,9-*def*]isoquinoline-1,3(2*H*)-dione (DBI)

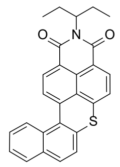

A solution of 2-methylnaphtho[1,2-*d*]thiazole (10 g, 50.2 mmol) in a mixture of ethylene glycol and aqueous 50% NaOH (v/v = 5/1, 120 mL) was refluxed under argon for 16 h before being poured into an ice-water bath and acidified to pH = 3 with 1M HCl solution. The organic phase was then extracted with CH_2Cl_2, dried over $MgSO_4$ and concentrated under reduced pressure. The resulting 1-aminonaphthalene-2-thiol was directly engaged in the next step without further purification. The latter was blended with 4-bromo-1,8-naphthalic anhydride (11.0 g, 39.7 mmol) and potassium carbonate (5.49 g, 39.7 mmol). DMF (270 mL) under air was then added and the reaction mixture was stirred for 16 h at room temperature. Isopentyl nitrite (15.7 mL, 119.1 mmol) was then added. An orange precipitate appeared upon stirring at 60 °C. After 24 h, the latter was filtrated, successively washed with water, methanol and finally dried. The resulting powder was directly blended added to a flask containing 3-aminopentane (5.33 mL, 45.7 mmol) and imidazole (77 g). This mixture was stirred for 16 h at 100 °C before being cooled down to room temperature. Then, a 1 M aqueous solution of HCl was gently added and the organic phase was subsequently extracted with CH_2Cl_2, dried over $MgSO_4$ and concentrated under reduced pressure. The crude was purified by column chromatography on silica gel (eluent: CH_2Cl_2) affording an orange solid (2.20 g, 10%). **^{1}H NMR** (300 MHz, $CDCl_3$): δ (ppm) 8.72 – 8.58 (m, 2H), 8.45 – 8.38 (m, 2H), 7.90 – 7.83 (m, 1H), 7.80 (d, J = 8.6 Hz, 1H), 7.59 – 7.50 (m, 3H), 7.39 (d, J = 8.6 Hz, 1H), 5.15 – 5.02 (m, 1H), 2.36 – 2.20 (m, 2H), 2.00 – 1.84 (m, 2H), 0.91 (t, J = 7.5 Hz, 6H). **^{13}C NMR** (75 MHz, $CDCl_3$): δ (ppm) 139.1, 136.8, 134.0, 133.5, 131.6, 130.4, 130.4, 129.2, 129.1, 127.5, 126.7, 125.9, 125.7, 124.0, 123.1, 121.1, 120.0, 118.6, 57.5, 25.1, 11.5. **HRMS** (MALDI): *m/z* calcd for $C_{27}H_{21}NO_2S$: 423.1288, found: 423.1293. Monocrystals were obtained by slow evaporation of chloroform.

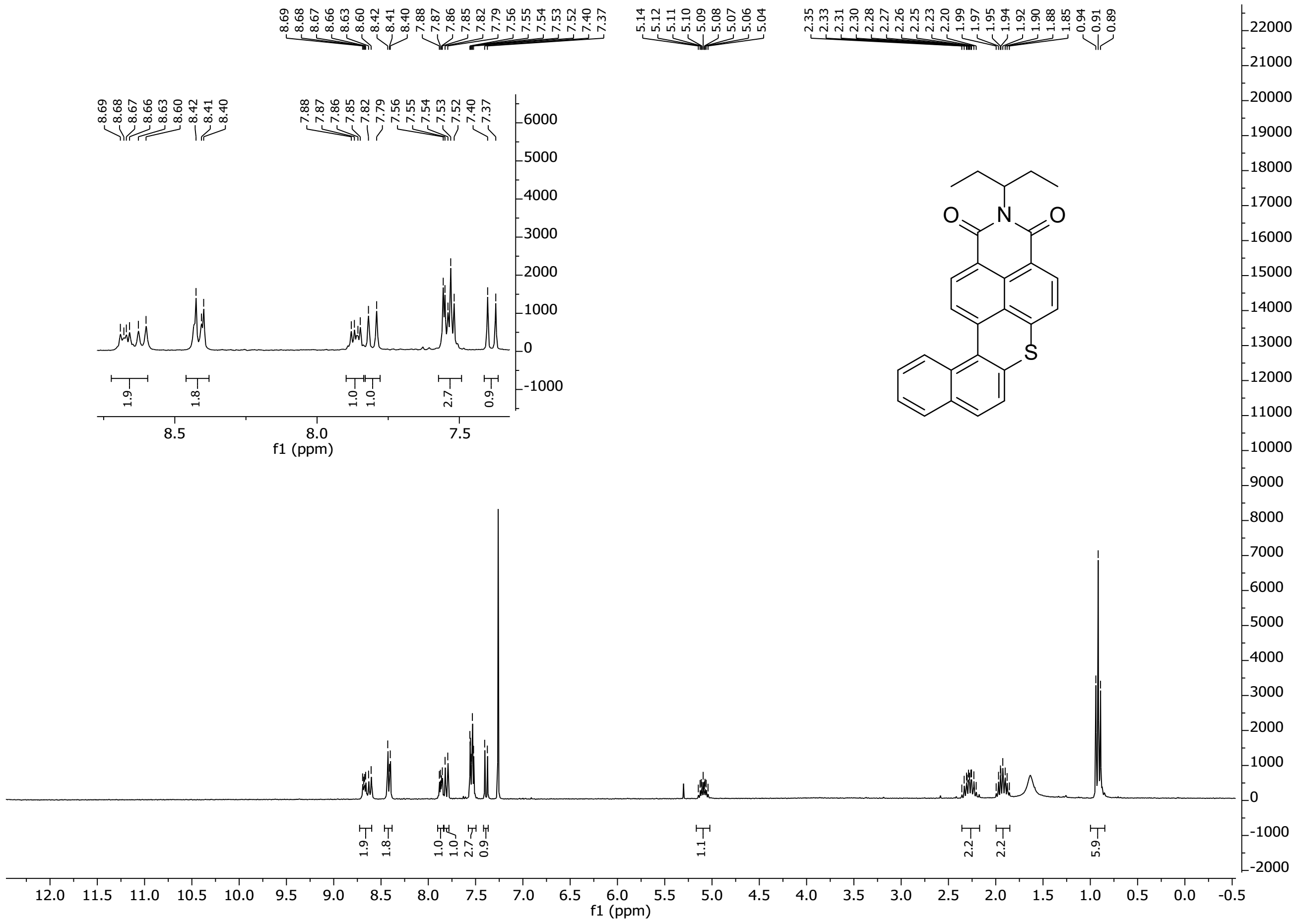

Supplementary Figure 1a. ^{1}H NMR (300 MHz, $CDCl_3$) spectrum of **DBI**.

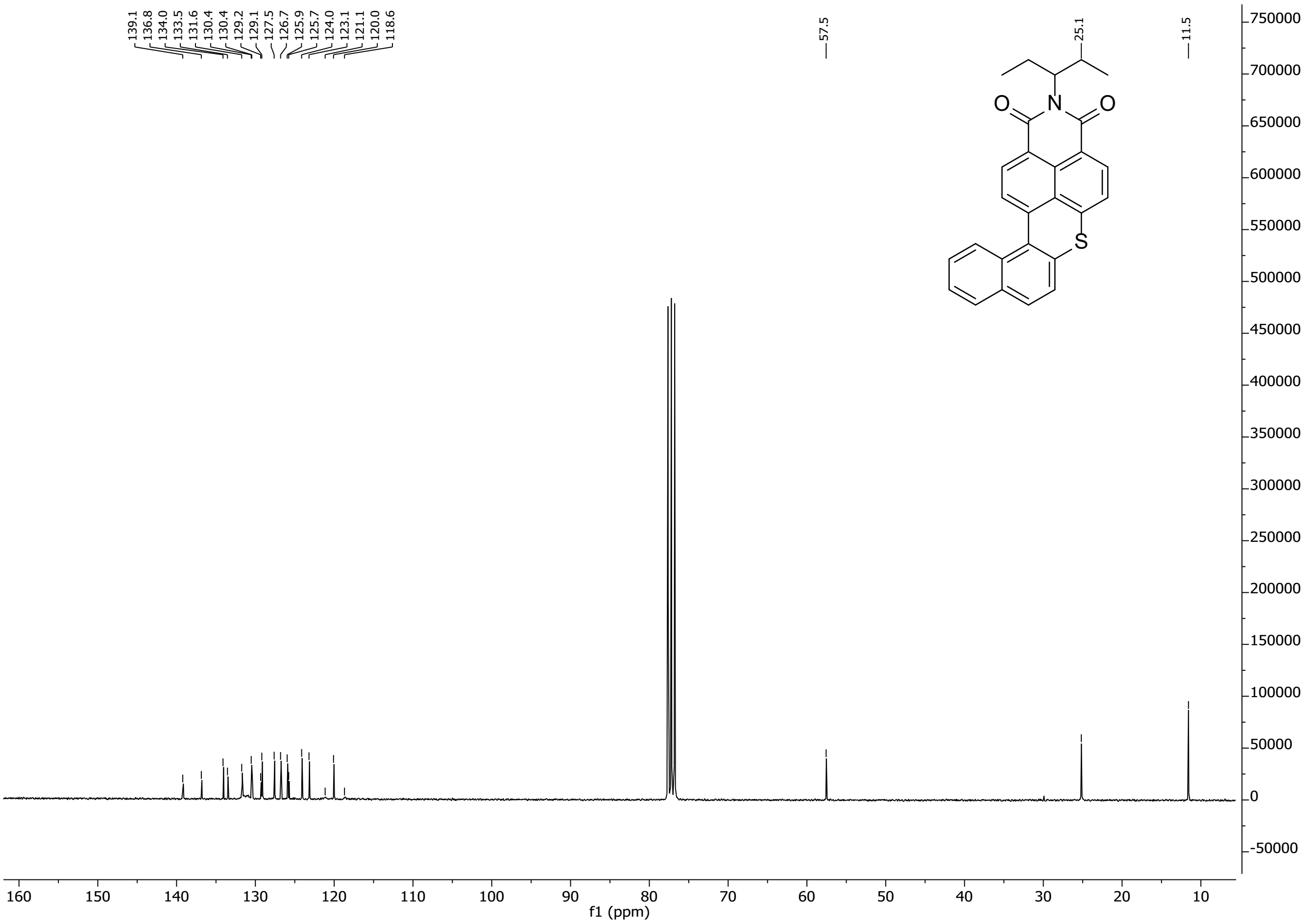

Supplementary Figure 1b. ^{13}C NMR (75 MHz, $CDCl_3$) spectrum of **DBI**.

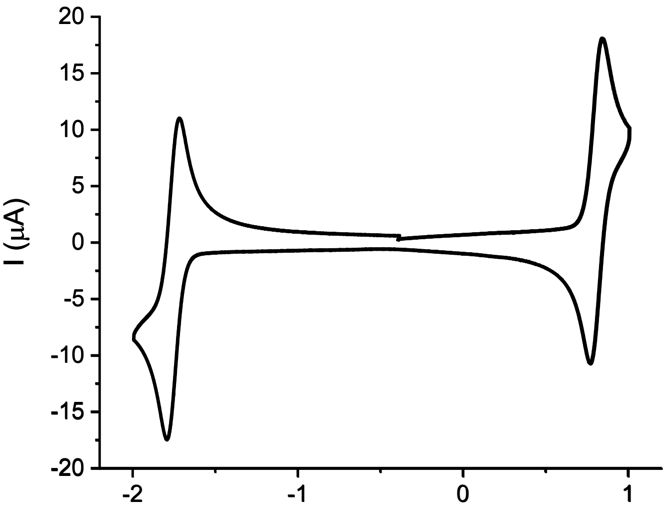

Supplementary Figure 2. Cyclic voltammograms of **DBI** (1 mM) in 0.1 M Bu_4NPF_6 in CH_2Cl_2 using a 100 mV/s scan rate.

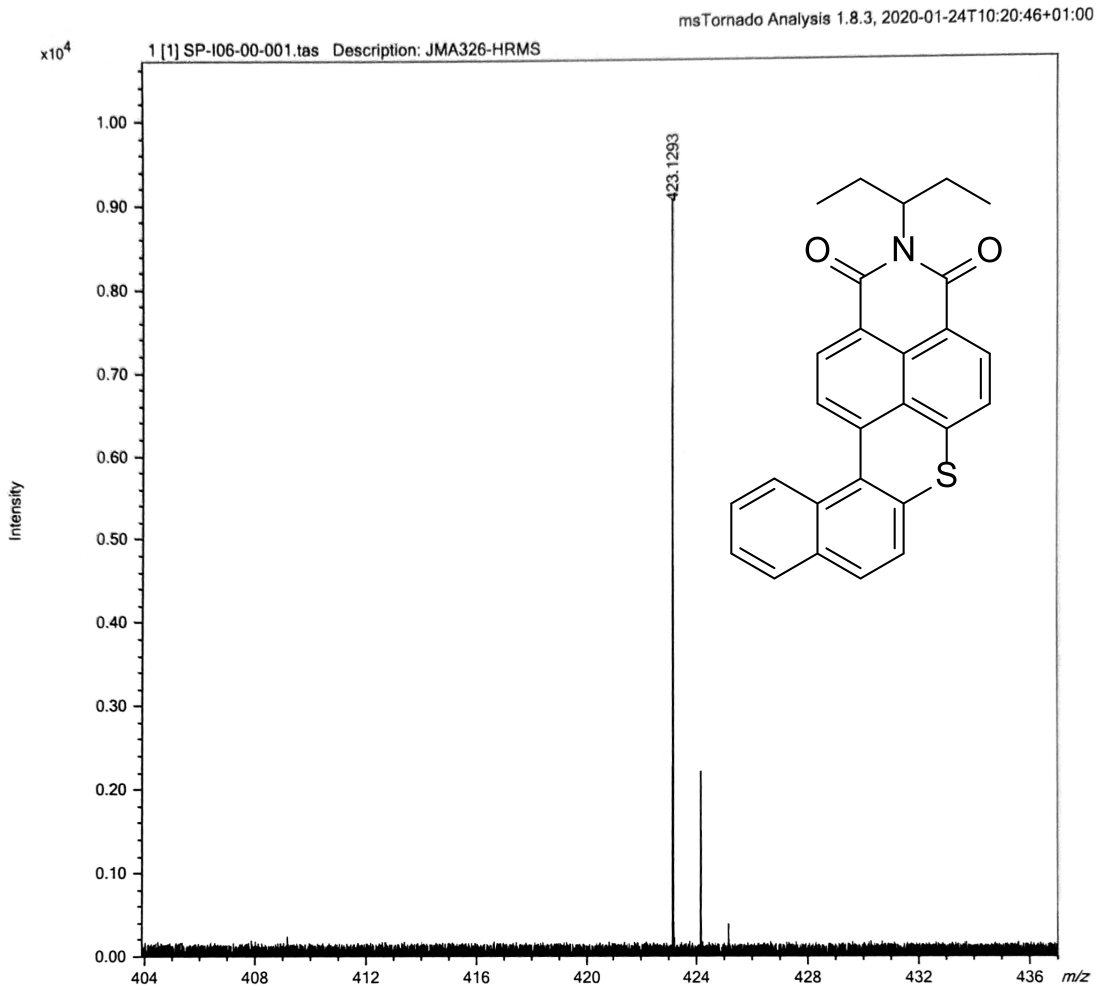

Elemental Composition Estimation

Parameters:

Mass	Tolerance	Electron Mode	Charge	DBE Range	Max Results
423.12934 ± 0.00127	3.0 ppm	Odd/Even	+1	-0.5 - 200.0	100

Elements

C	0 - 27	H	0 - 40	N	0 - 5	O	0 - 5	F	0 - 0	Cl	0 - 0	S	0 - 1

Results:

#	Formula	Mass	DBE	Abs. Error (u)	Error (u)	Error (ppm)
1	C27 H21 N O2 S	423.12875	18.0	0.00059	0.00059	1.39

Supplementary Figure 3. HRMS (MALDI) spectrum of **DBI**: *m/z* calcd for $C_{27}H_{21}NO_2S$: 423.1288, found: 423.1293.

Supplementary Table 1. Crystal data collection and refinement parameters of **DBI**.

Molecule	**DBI**
Empirical formula	$C_{27}H_{21}NO_2S$
Formula weight	423.51
Temperature (K)	150.0(1)
Wavelength(Å)	1.54184
Crystal system, space group	Orthorhombic, P 21 21 21
a(Å)	7.4412(2)
b(Å)	8.0589(3)
c(Å)	33.5265(13)
α(deg)	90
β(deg)	90
γ(deg)	90
Z	4
Volume (Å^3)	2010.51(12)
Calculated density (Mg/m^3)	1.399
Absorption coefficient (mm^{-1})	1.630
θ range (deg)	2.636 to 72.412
Completeness to θ = 70.000	98.5%
GOF	1.130
Largest diff. peak & hole (e/Å^3)	0.486 & -0.400

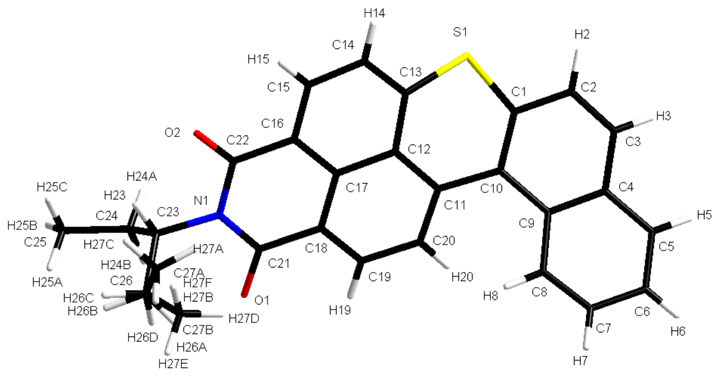

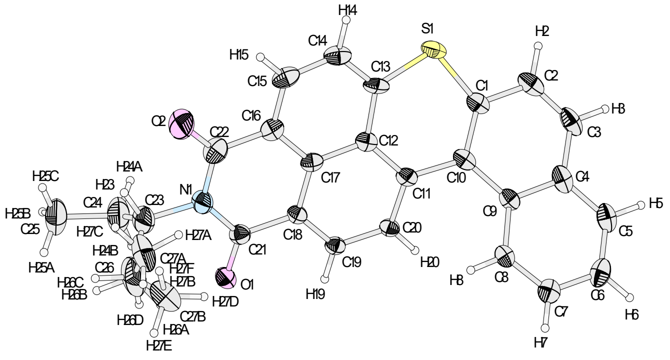

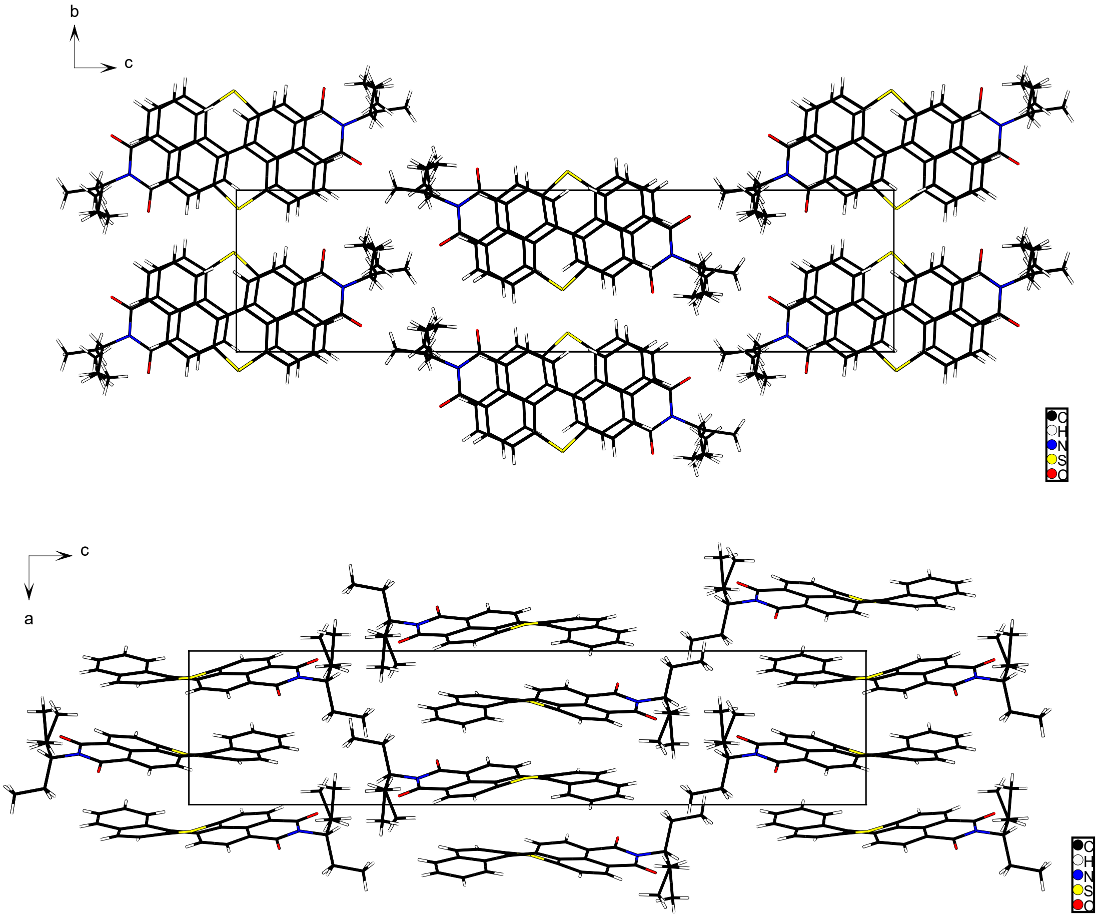

Supplementary Figure 4. Molecular structures of **DBI** determined single crystal X-ray diffraction.

Spin-Orbit Coupling calculations

Ground and excited states geometry optimizations were carried out with the Gaussian16 code.(1) The global hybrid functional PBE0 was used both for ground state and excited state geometry optimisations.(2) This functional was chosen because of its accuracy to reproduce spectroscopic properties of benzothioxanthene imide derivatives.(3) Structural optimisations and subsequent frequency calculations for both the ground and excited states were performed using an all electron Pople triple zeta basis set with one polarisation function on all atoms and one diffuse function of heavier atoms, known as 6-311+G(d,p), for H, C, N, O and S atoms.(4) Bulk solvent effects were included using the Polarizable Continuum Model (PCM) of Tomasi and co-workers.(5) Default radii (from the UFF, scaled by 1.1) were used. Excited state geometry was obtained by minimizing the forces of the S_1 state computed at the TD-DFT level by considering the 3 first excited states. The Dalton(6) program was used to compute the SOC between the three first triplet states (namely T_1, T_2 and T_3) and the S_1 state at the S_1 optimized geometry using the quadric-response TD-DFT at the CAM-B3LYP/PCM level with the cc-pVDZ basis set adapted for the Douglas-Kroll calculations.(7) The Spin-Orbit Coupling was computed using the Douglas-Kroll Hamiltonian along with the spin-orbit mean field approach.(8)

Absorption, emission and phosphorescence spectral signatures of DBI

Absorption spectra were recorded on a JASCO V-650 spectrophotometer in diluted solution (ca. 10^{-5} or 10^{-6} M), using spectrophotometric grade solvents. Emission spectra were measured using Horiba-Jobin–Yvon Fluorolog-3 fluorimeter. The steady-state luminescence was excited by unpolarised light from a 450 W xenon continuous wave (CW) lamp and detected at an angle of 90° for measurements of dilute solutions (10 mm quartz cuvette) by using a Hamamatsu R928. Spectra were corrected for both excitation source light-intensity variation and emission spectral responses. 77K time-gated phosphorescence was measured with a 50 µs delay.

A summary of the main spectroscopic and photophysical data for the **DBI** is included in Supplementary Table 2.

Supplementary Table 2. DBI photophysical data.

λ_{max} (nm)	λ_{em} (nm)	Stoke's Shift (cm^{-1})	Φ_F[a]	Φ_Δ[b]	$E_{Triplet}$ (cm^{-1})
483	544	2100	0.08	0.95	14,250

[a] Measured using Coumarin-153 as reference (Φ_F = 0.45 in methanol).

[b] Measured using Phenalenone as reference (Φ_F = 0.95 in dichloromethane).

Fluorescence quantum yield

Luminescence quantum yields (Φ_F) were measured in diluted solutions with an absorbance lower than 0.1, by using the following Equation 1:

$$\frac{\Phi_{F\,(x)}}{\Phi_{F\,(r)}} = \frac{A_r}{A_x} \times \frac{n_x^2}{n_r^2} \times \frac{D_x}{D_r} \qquad (Eq.1)$$

where A is the absorbance (or optical density) at the excitation wavelength, *n* the refractive index of the solvent and D the integrated luminescence intensity. "r" and "x" stand for reference and sample, respectively. Here, the reference is coumarin-153 in methanol (Φ_F = 0.45). Excitations of reference and sample compounds were performed at the same wavelength. The reported results are the average of 4-5 independent measurements at various absorbances (comprised between 0.01–0.1) for both sample and reference. The plot of the integrated luminescence intensity *vs.* absorbance gives straight line with excellent correlation coefficients and the slope S can be determined for both sample (x) and reference (r). Equation 1 becomes Equation 2.

$$\frac{\Phi_{F\,(x)}}{\Phi_{F\,(r)}} = \frac{S_x}{S_r} \times \frac{n_x^2}{n_r^2} \qquad (Eq.2)$$

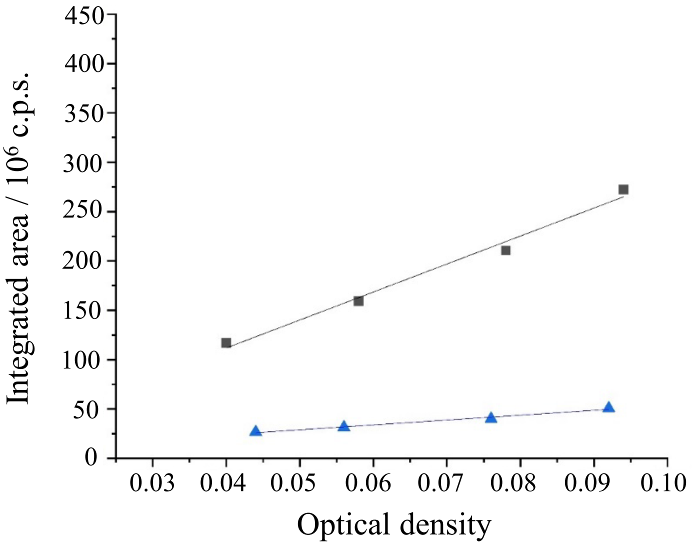

Supplementary Figure 5. Integrated emission area according to optical density of Coumarine-153 (black squares) and **DBI** (blue triangles) in dichloromethane at room temperature.

Determination of singlet oxygen quantum yield

Singlet oxygen quantum yield determination Φ_Δ was calculated based on the following equation:

$$\frac{\Phi_{\Delta\,(x)}}{\Phi_{\Delta\,(r)}} = \frac{S_x}{S_r} \times \frac{n_x^2}{n_r^2} \qquad (Eq.3)$$

The reported results are the average of 4-5 independent measurements at various absorbances (comprised between 0.01–0.1) for both sample and reference. The plot of the integrated singlet oxygen luminescence intensity *vs.* absorbance gives straight line with excellent correlation coefficients. The reference used is phenalenone (Φ_Δ = 0.98 in dichloromethane).

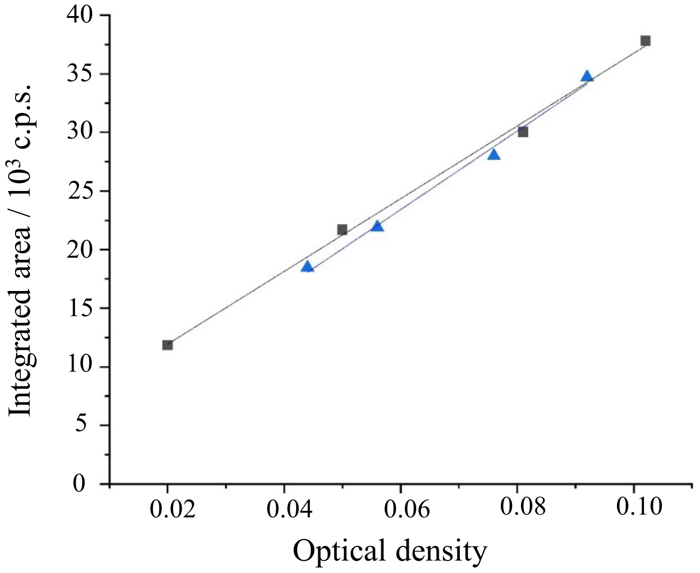

Supplementary Figure 6. Integrated singlet oxygen emission area according to optical density of phenalenone (black squares) and **DBI** (blue triangles) in dichloromethane at room temperature.

Blue light-induced morphological changes of DBI-treated HeLa cells

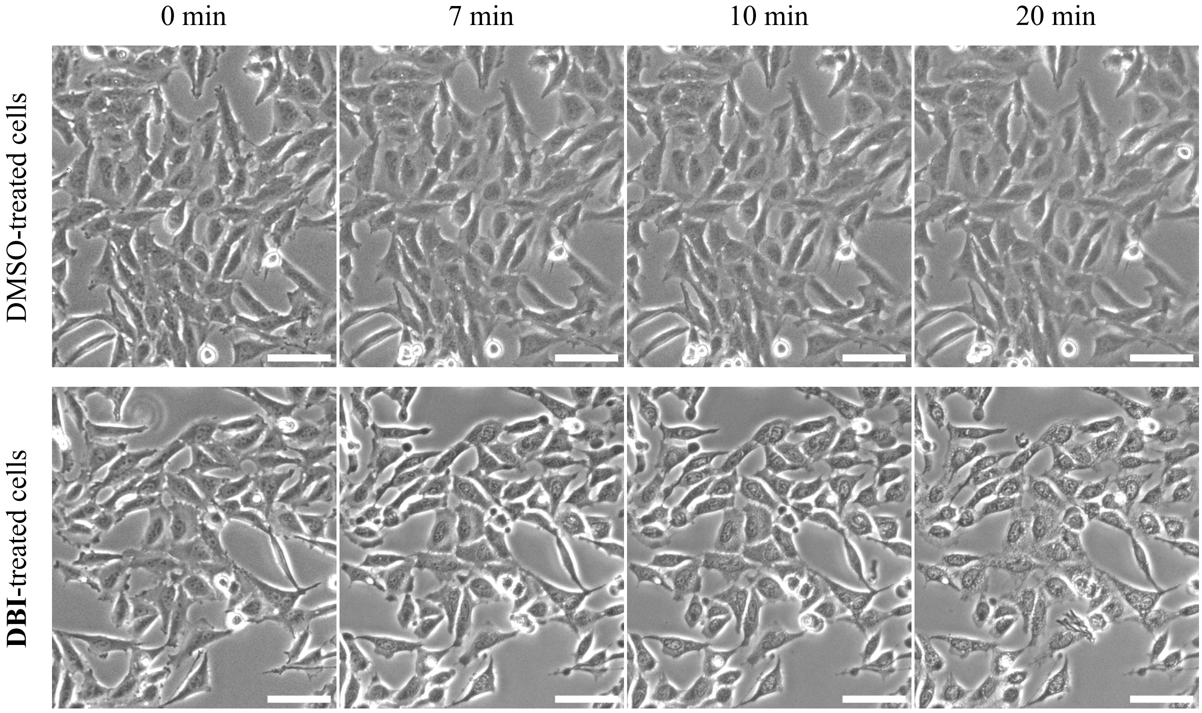

Supplementary Figure 7. Time-lapse experiments of live HeLa cells. Light-induced morphological changes of **DBI**-treated (1 μM) HeLa cells after 0, 7, 10 and 20 min of blue light irradiation. Control experiments were performed in DMSO-treated HeLa cells.

Dark and photo-cytotoxicity of Temoporfin

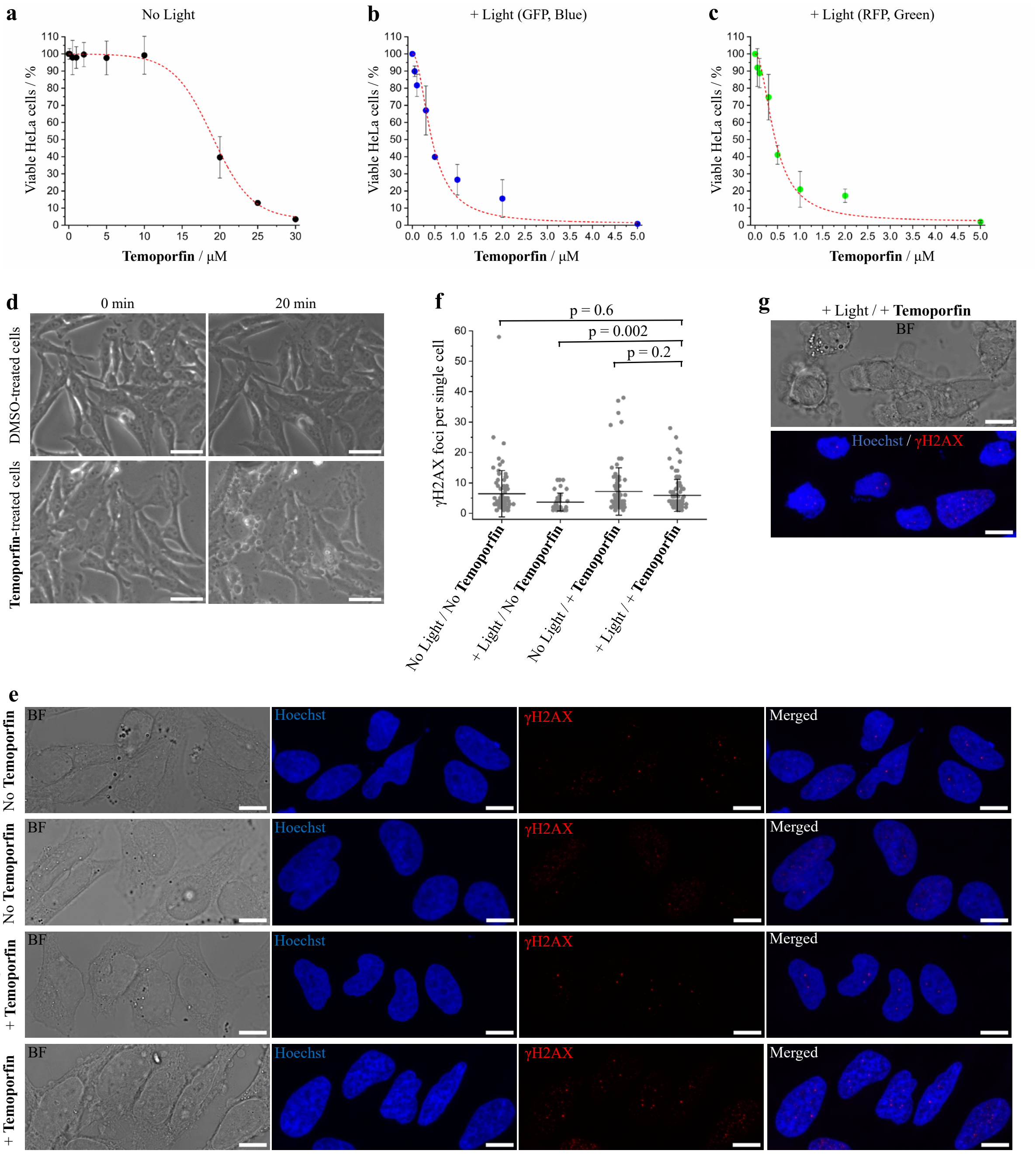

Supplementary Figure 8. a) Dark cytotoxicity of Temoporfin on HeLa cells. **b-c)** Photo-cytotoxicity of Temoporfin on HeLa cells in the presence of blue (27 mW cm^{-2}) or green (10 mW cm^{-2}) light irradiated for 6 min. Error bars indicate mean ± SD (n = 3). **d)** Light-induced morphological changes of Temoporfin-treated (10 µM) HeLa cells after 0 and 20 min of blue light irradiation. Control experiments were performed in DMSO-treated HeLa cells. **e)** Immunodetection of γH2AX in HeLa cells treated either with Temoporfin (5 µM) or DMSO (0.05 % v/v) for 24 h. The cells were irradiated with blue light using a LED light cube (30 mW cm^{-2}) for 20 min and incubated for an additional 30 min at 37 °C before PFA-fixation. The control cells were not irradiated but otherwise treated as described above. HeLa cells were co-stained with the nuclear dye Hoechst 33342 (500 nM, blue signal). $\lambda_{exc}/\lambda_{em}$: 405/440-460 nm for Hoechst (blue signal) and 598/620-750 for γH2AX (red signal). Scale bar 10 µm. **f)** Quantification of

γH2AX nuclear foci in the experimental conditions provided in (e). Data represent populations of individual cells (N = from 42 to 92 cells per condition). Error bars indicate mean ± SD. Analysis of the data was performed using a two-sample *t* test and the *p* value is indicated. **g)** Representative image of immunodetection of γH2AX, performed as in (e), but focusing on cells that showed a dramatic change in cell morphology upon both Temoporfin (5 μM) and light treatment.

DBI co-localized with CD63 signal

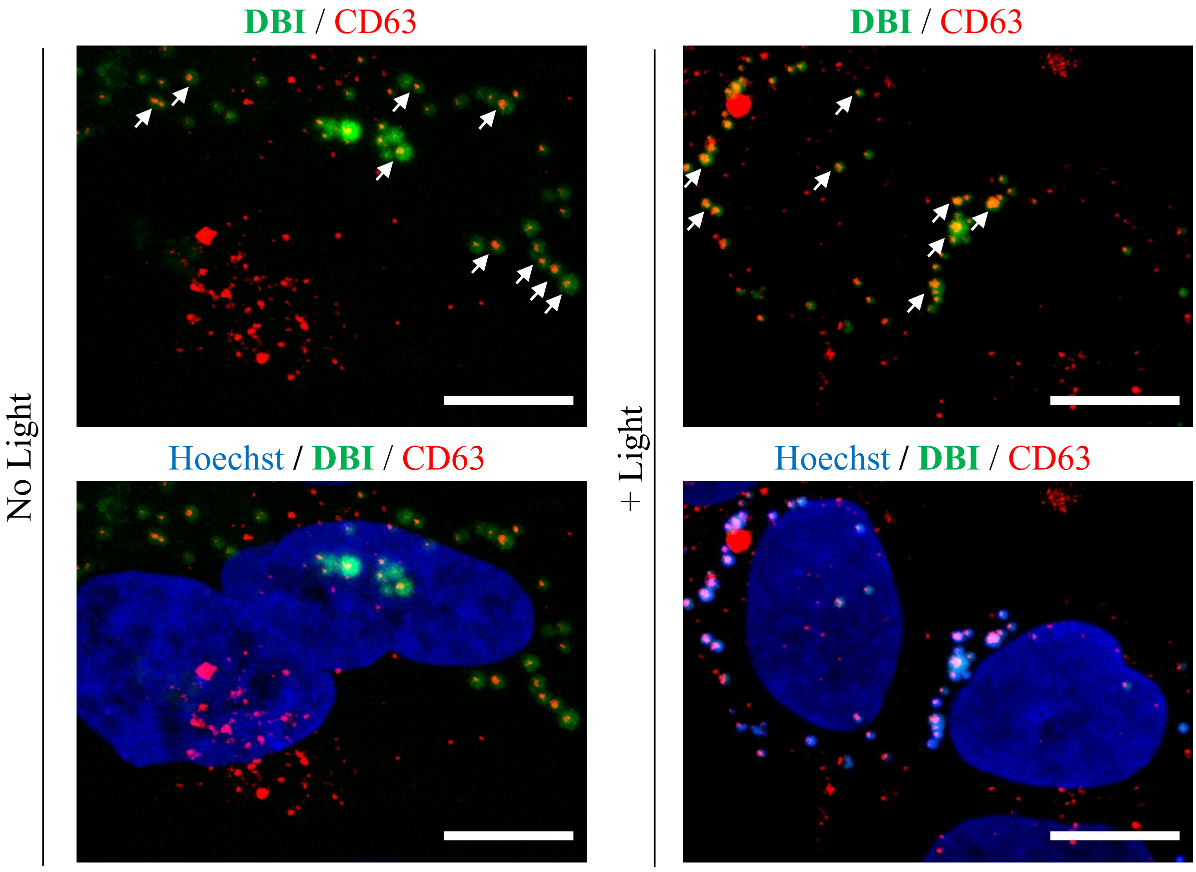

Supplementary Figure 9. Enlargement of Fig. 3b-c to show the co-localized **DBI** and CD63 signals. White arrows in the upper figure panel aim to show the co-localized **DBI** and CD63 signals. Immunofluorescence experiments of HeLa cells treated with **DBI** (1 μM) for 24 h and either non-irradiated or irradiated with a blue LED light cube (30 mW cm^{-2}) for 20 min and incubated for additional 30 min at 37 °C before paraformaldehyde (PFA) fixation. $\lambda_{exc}/\lambda_{em}$: 405/440-460 nm for Hoechst (blue signal); and 528/540-590 nm for **DBI** (green signal); and 598/620-750 nm for anti-CD63 (red signal). Scale bar 10 μm.

DBI co-localized with Hoechst signal

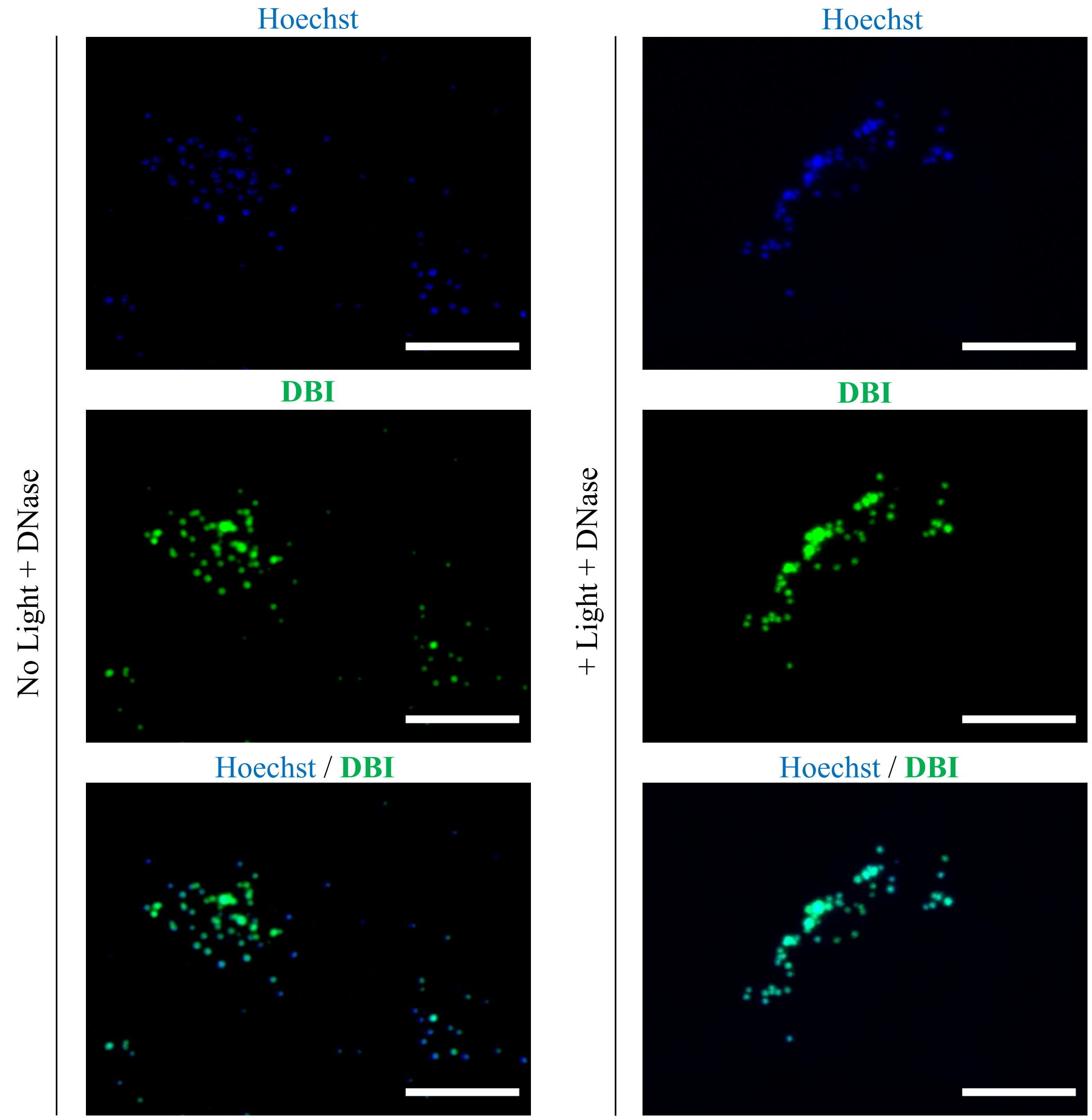

Supplementary Figure 10. Enlargement of Fig. 3d to show the co-localized **DBI** and Hoechst signals. Confocal fluorescence images of HeLa cells treated with **DBI** (1 µM) for 24 h and either non-irradiated or irradiated with blue light using a LED light cube (30 mW cm^{-2}) for 20 min and incubated for additional 30 min at 37 °C before paraformaldehyde (PFA) fixation. After cell fixation, DNase I was added to the cells that were incubated at 37 °C for 24 h. Finally, Hoechst 33342 (500 nM) was used to detect DNA in cells. $\lambda_{exc}/\lambda_{em}$: 405/440-460 nm for Hoechst (blue signal); and 528/540-700 nm for **DBI** (green signal). Scale bar 10 µm.

In vitro G4 binding studies

Materials: Solvents, reagents, chemicals and biological templates were purchased from commercial suppliers (Sigma-Aldrich and Eurofins Genomics) and used without further modifications unless otherwise stated. Oligonucleotides were diluted with ultrapure water (DNase and RNase free) and stored at 5 °C. RNA oligos were diluted in DEPC-treated water. The exact oligonucleotide concentration was determined by UV/Vis spectroscopy using the molar extinction coefficients (ε_{260}) provided in the Supplementary Table 3 and calculated by using an oligo analyzer on the IDT website. An aqueous stock solution (1 M) of TRIS buffer was prepared by dissolving tris(hydroxymethyl)aminomethane in water and the pH was then adjusted to 7.2.

General procedures: Steady-state emission spectra were recorded with a Jasco FP-6500 spectrofluorometer equipped with a JascoPeltier-type temperature controller (ETC2736, at Umeå University). 1.0 cm path length quartz cells were used throughout these measurements. Fluorescence lifetime decays were collected via Time-Correlated Single Photon Counting (TCSPC) setup by using a Fluorolog TCSPC (Horiba Jobin Yvon). The excitation source was a NanoLED with excitation peak at 490 nm and a long pass filter (>500 nm) was inserted into the excitation path. CD spectra were recorded with a Jasco J-1700 CD spectrometer or with a Jasco J-720 spectropolarimeter equipped with a JascoPeltier-type temperature controller (PTC-423L). Fluorescence polarization was recorded with a Biotek Synergy H4.

Oligonucleotide folding: Oligonucleotides were heated at 95 °C for 5 min in the presence of 100 mM KCl and then slowly allowed to reach room temperature overnight. The list of the oligonucleotides used in this study is provided in the Supplementary Table 3.

G4-binding screening: A solution of **DBI** (1.0 μM, DMSO 0.1 % v/v) was prepared by diluting the stock solution (**DBI**: 5mM in DMSO) in a suitable amount of buffered water (KCl = 100 mM, TRIS buffer 50 mM, pH = 7.2). The freshly prepared **DBI** solutions were then titrated with the annealed oligonucleotide solutions (5.0 μM) and left to equilibrate for 30 min before recording the emission spectra. Analysis and data representation were performed by using OriginPro 2020 software.

Steady-state and time-resolved fluorescence titrations: DBI (1.0 μM, DMSO 0.1 % v/v) was titrated with incremental additions of *HIF-1α*, mut *HIF-1α* or C-rich *HIF-1α* (from 0 to 5.0 equivalents). The emission spectra were recorded in the 525-700 wavelength range upon excitation at 520 nm. The turn-on fluorescence response, reported in Figure 4a, of **DBI** complexed with various oligonucleotides was performed by dividing the emission of the complex (F) over the emission of **DBI** alone (F_0) at 561 nm. Data representation was performed by using OriginPro 2020 software. Lifetimes were obtained by using bi-exponential (**DBI**-*HIF-1α*) or tri-exponential (**DBI**, **DBI**-mut *HIF-1α* and **DBI**-C-rich *HIF-1α*) decay functions. Then the averaged fluorescence lifetimes (τ_{ave}) were calculated according to the following equation:

$$\tau_{ave} = \frac{\sum A_i \tau_i^2}{\sum A_i \tau_i} \qquad (Eq. 4)$$

where A_i is the i-th relative amplitude and τ_i is the i-th component of fluorescence lifetime, respectively.

Fluorescence polarization binding assay: DBI (1.0 μM, DMSO 1 % v/v) was titrated with incremental additions of oligonucleotides ranging from 0 to 10 equivalents. The binary mixtures (**DBI**-oligonucleotide) were left to equilibrate for 1 hour before recording the signal. The variation in the fluorescence polarization signal of **DBI** was monitored upon the addition of incremental oligonucleotide concentrations and in certain systems provides typical saturation binding curves. Binding constants were obtained with Bindfit(9,10) by using multiple global fitting methods (Nelder–Mead method). Data representation was performed by using OriginPro 2020 software.

^{1}H NMR G4 studies. *HIF-1α* and mut *HIF1α* were dissolved at a concentration of 110 µM in KCl 100 mM and 10 mM Tris buffer (pH = 7.2). 10% D_2O was added to the solutions. All spectra were recorded at 298 K on a Bruker 850 MHz Avance III HD spectrometer equipped with a 5 mm TCI cryoprobe. Excitation sculpting was used in the 1H NMR titration experiments, and 256 scans were recorded. Processing was performed in Topspin 3.6 (Bruker Biospin, Germany).

CD-based thermal melting assays. CD melting data were acquired by increasing the temperature and monitoring the characteristic CD peaks of each sequence used. The oligonucleotide concentration was 2 µM and **DBI** concentration was 10 µM. Experiments were performed in TRIS buffer (10 mM, pH = 7.2) and KCl (5 mM). Melting values were estimated by fitting the normalized melting curves with a dose response function using OriginPro 2020 software.

Supplementary Table 3. List of the biologically relevant natural and synthetic DNA and RNA oligonucleotides used for biophysical and biochemical studies.

Name	Sequence[a]	Length bp	Absorptivity[b] M^{-1} cm^{-1}	GC %	Topology
HIF-1α	GCGCGGGGAGGGGAGAGGGGGCGGGAGCGCG	31	309400	87.1	parallel
HIF-1α no flanking residues	GGGGAGGGGAGAGGGGGCGGG	21	219300	85.7	parallel
c-MYC Pu22	TGAGGGTGGGTAGGGTGGGTAA	22	228700	59.1	parallel
VEGF	GGGAGGGTTGGGGTGGG	17	171400	76.5	parallel
VAV-1	GGGCAGGGAGGGAACTGGG	19	194700	73.7	parallel
c-KIT 2	CCCGGGCGGGCGCGAGGGAGGGGAGG	26	253400	88.5	parallel
*CEB*25	AGGGTGGGTGTAAGTGTGGGTGGGT	25	253100	60.0	parallel
BCL-2	GGGCGCGGGAGGGAATTGGCGGGG	24	237400	79.2	parallel
c-MYC Pu24T	TGAGGGTGGTGAGGGTGGGGAAGG	24	248200	66.7	parallel
4G3U3	GGGUUUGGGUUUGGGUUUGGG	21	185600	57.1	parallel
TERRA	UUAGGGUUAGGGUUAGGGUUAGGG	24	235500	50.0	parallel
FMR1	GGAGGGGGAGGAAGAGGACAAGGAGGAAGAGG	32	354500	62.5	parallel
Tel-22	AGGGTTAGGGTTAGGGTTAGGG	22	228500	54.5	hybrid
Bom17	GGTTAGGTTAGGTTAGG	17	174600	47.1	antiparallel
TBA	GGTTGGTGTGGTTGG	15	143300	60	antiparallel
mut *HIF-1α*	GCGCGGCGAGCGGAGAGGGCGCGCGAGCGCG	31	295400	87.1	non-G4
C-rich *HIF-1α*	CGCGCTCCCGCCCCCTCTCCCCTCCCCGCGC	31	238500	87.1	ssDNA
scr *ss*-DNA	GGATGTGAGTGTGAGTGTGAGG	22	227000	54.5	ssDNA
sc *ds*-DNA	CAATCGGATCGAATTCGATCCGATTG	26	253200	46.2	*ds*-DNA
sc *ds*-RNA	CAAUCGGAUCGAAUUCGAUCCGAUUG	26	244100	46.2	*ds*-RNA
ds-DNA *HIF-1α* no flanking res.	GGGGAGGGGAGAGGGGGCGGG<br>CCCGCCCCCTCTCCCCTCCCC	21<br>21	219300<br>156400	85.7	*ds*-DNA[c]
ds-DNA *HIF-1α*	GCGCGGGGAGGGGAGAGGGGGCGGGAGCGCG<br>CGCGCTCCCGCCCCCTCTCCCCTCCCCGCGC	31<br>31	309400<br>238500	87.1<br>87.1	*ds*-DNA[c]

[a] Conventional 5′ to 3′ direction. [b] Molar extinction coefficient calculated by using oligo analyzer on the IDT web site. [c] *ds*-DNA sequences are formed by mixing an equimolar concentration of the two strands.

Supplementary Table 4. Forward (F) and reverse (R) primer sequences of different oncogene promoters and housekeeping genes used in qPCR.

	Primer sequences (5´ - 3´)
hTERT-F	CAGCGCTGCCTGAAACTC
hTERT-R	GTCCTGCCCCTTCACCTT
HIF-1α-F	GCCTCCTGATTGGCTGAGA
HIF-1α-R	CACTGTGCACTGAGGAGCTG
c-MYC-F	AAGGGAGAGGGTTTGAGAGG
c-MYC-R	CGGAGATTAGCGAGAGAGGA
45S rDNA-1196-F	GTCGTGTGTGGGTTGACTTC
45S rDNA-1196-R	GCGGTACGAGGAAACACCT
45S rDNA-6374-F	GTCGGGTGGGGGCTTTAC
45S rDNA-6374-R	CCAAGAGGAGAGGGGGTTG
GAPDH promoter-F	TCCAATTCCCCATCTCAGTC
GAPDH promoter-R	TAGTAGCCGGGCCCTACTTT
GAPDH-F	CTCTGCTCCTCCTGTTCGAC
GAPDH-R	ACGACCAAATCCGTTGACTC

CD spectral signatures

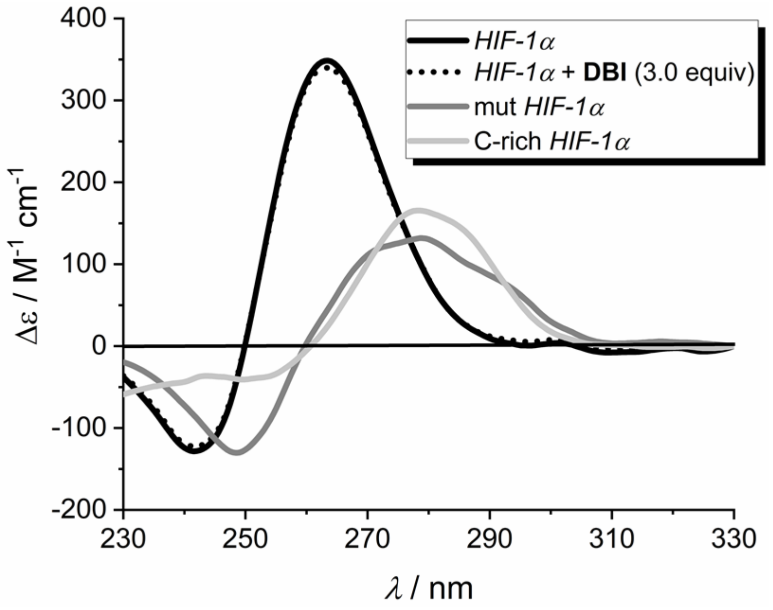

Supplementary Figure 11. CD spectra of *HIF-1α*, mut *HIF-1α* or C-rich *HIF-1α*. Only *HIF-1α* template fold into a parallel G4 conformation as highlighted by the appearance of positive and negative peaks centered at ~ 264 and 240 nm, respectively. (*HIF-1α*, mut *HIF-1α* or C-rich *HIF-1α* = 1.0 µM, **DBI** = 3.0 µM, KCl = 100 mM and Tris = 10 mM pH 7.2).

^{1}H NMR spectra in the imino-proton region for *HIF-1α* and mut *HIF-1α*

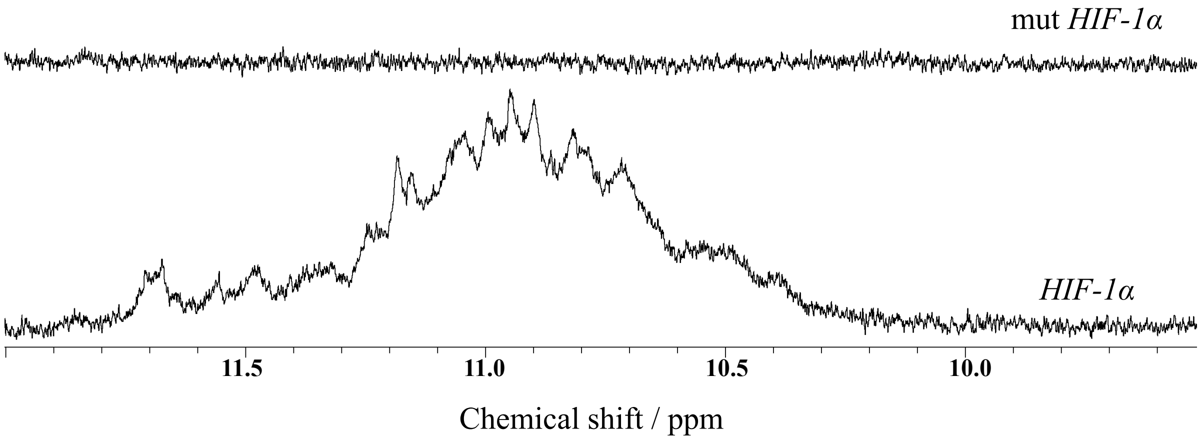

Supplementary Figure 12. ^{1}H NMR spectra in the imino-proton region for *HIF-1α* and mut *HIF-1α* (G4 template = 100 µM, TRIS buffer 10 mM, pH = 7.2 and KCl 100 mM). The absence of imino-proton signals in the mut *HIF-1α* spectrum indicates the absence of a G4 structure.

Fluorescence titration studies of DBI complexed with C-rich *HIF-1α* or sc *ds*-DNA

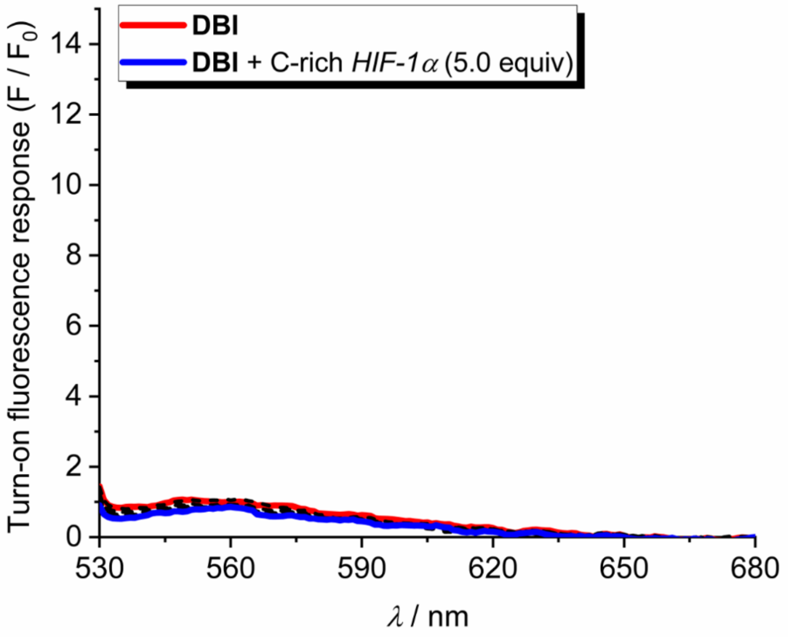

Supplementary Figure 13. Steady-state emission spectra of **DBI** (1 µM, DMSO 0.1 % v/v) complexed with C-rich *HIF-1α* (from 0 to 5µM) in TRIS buffer (50 mM, pH = 7.2) and KCl (100 mM). $\lambda_{exc}/\lambda_{em}$: 520/525-700 nm.

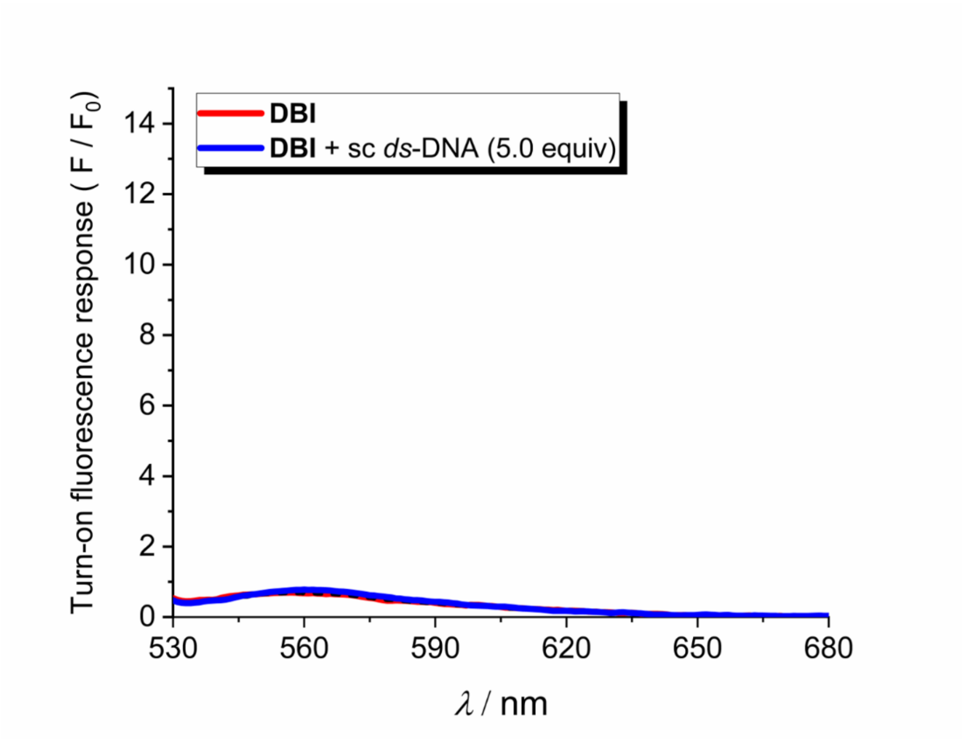

Supplementary Figure 14. Steady-state emission spectra of **DBI** (1 µM, DMSO 0.1 % v/v) complexed with sc *ds*-DNA (from 0 to 5µM) in TRIS buffer (50 mM, pH = 7.2) and KCl (100 mM). $\lambda_{exc}/\lambda_{em}$: 520/525-700 nm.

Viscosity-dependent studies

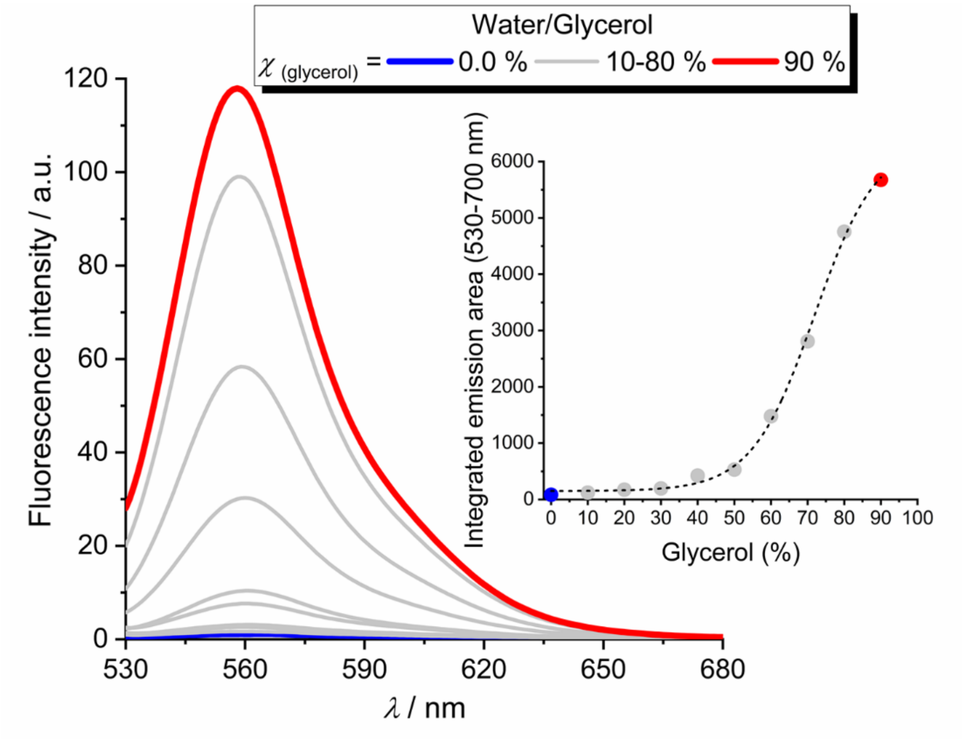

Supplementary Figure 15. Viscosity-dependent emission spectra of **DBI** (2 µM) at different water to glycerol content. $\lambda_{exc}/\lambda_{em}$: 520/525-700 nm.

Fluorescence polarization

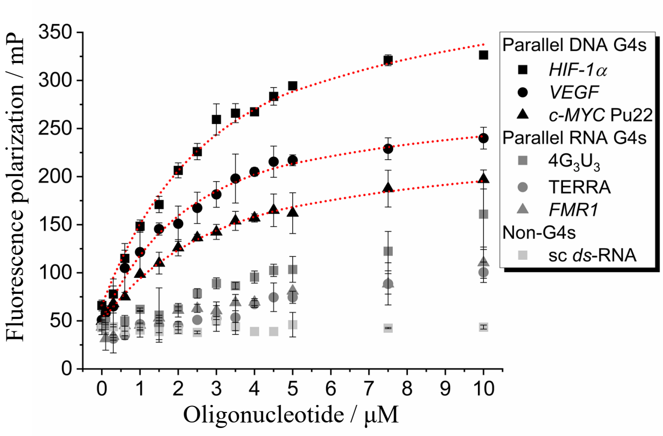

Supplementary Figure 16. Fluorescence polarization studies of **DBI** (1 µM, DMSO 1 % v/v) in the presence of parallel DNA G4s as well as RNA G4s and a self-complementary (sc) *ds*-RNA (from 0 to 10 µM) in TRIS buffer (50 mM, pH = 7.2) and KCl (100 mM). $\lambda_{exc}/\lambda_{em}$: 485 (±20)/620 (±40) nm. A superimposed dashed line in the **DBI**-*HIF-1α*, **DBI**-*VEGF* and **DBI**-*c-MYC* Pu22 systems is the result of nonlinear fitting with a 1:1 binding model. Data for **DBI**-*HIF-1α*, **DBI**-*VEGF* and **DBI**-*c-MYC* Pu22 are also shown in Figure 4f in the main manuscript. No quantitative data analysis was performed on **DBI** complexed with RNA G4s or sc *ds*-RNA due to the absence of a well-defined optical response.

CD-melting profiles

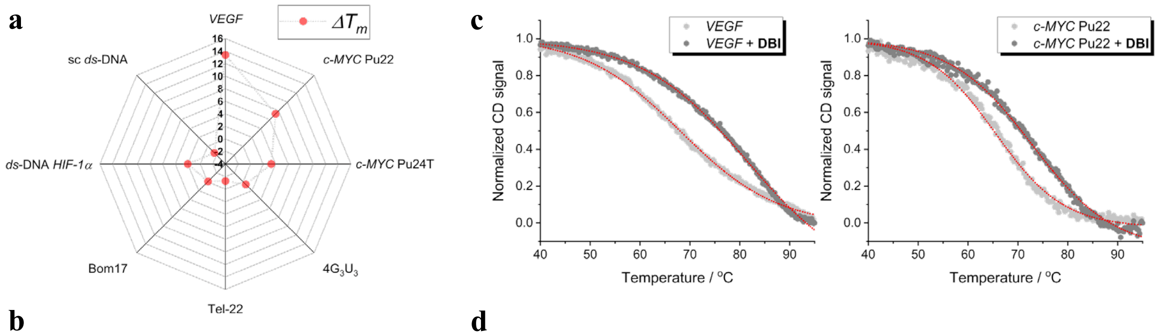

b

System	ΔT_m / °C
VEGF-**DBI**	13.4
c-MYC Pu22-**DBI**	7.3
c-MYC Pu24T-**DBI**	3.3
$4G_3U_3$ -**DBI**	0.6
Tel-22-**DBI**	-1.3
Bom17-**DBI**	-0.1
ds-DNA *HIF-1α*-**DBI**	2
sc *ds*-DNA-**DBI**	-1.5

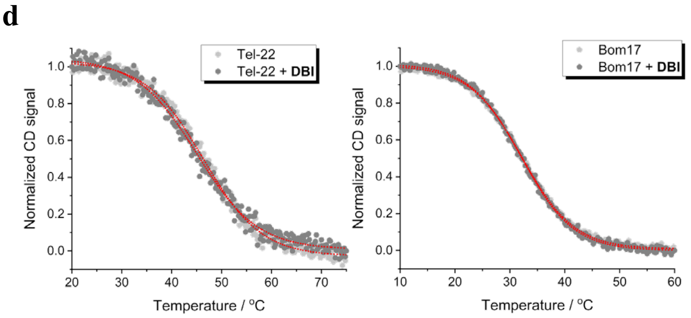

Supplementary Figure 17. a) The radar graph shows the extent of thermal stabilization (ΔT_m) induced by **DBI** on a panel of DNA and RNA G4s as well as DNA duplexes. **b)** ΔT_m induced by **DBI** to G4 and duplex templates. **c-d)** Examples of melting profiles with parallel (c), hybrid and antiparallel (d) G4s. **DBI** (10 µM, DMSO 2 % v/v), oligonucleotides (2 µM) in TRIS buffer (10 mM, pH = 7.2) and KCl (5 mM).

Molecular Dynamics simulations

Molecular dynamics simulations of G4s in the presence of two **DBI** molecules were performed using Amber20 (see Supplementary Table 5). The initial position of the **DBI** ligands mimics the binding pose of BMVC molecules in 6O2L structure.(11) Two stacked positions are modeled for each ligand, providing different environment around the rings and the sulfur atom. Parmbsc1(12) and Gaff(13) parameters were used for G4 nucleobases and **DBI** respectively. In addition, **DBI** atomic charges were determined using RESP(14) approach at B3LYP/6-31+G(d,p)/IEFPCM(15) level of theory using Gaussian09.

Each system is solvated in a rectangular-shaped TIP3P water box to ensure a minimum distance of 12 Å between the solute and the edge of the periodic box. K^+ and Cl^- ions are added to neutralize the boxes and to reach an ionic concentration of about 0.1 M. After minimization of the systems, all the simulations were run using a timestep of 2 fs in combination with the SHAKE algorithm constraining the hydrogen covalent bonds, a 10 Å cutoff for intermolecular forces and the Particle Mesh Ewald method for electrostatic long-range interactions. We start with a heating procedure of 60 ps to increase the temperature from 0 to 300 K in the NVT ensemble, followed by a 1 ns equilibration step in the NPT ensemble with a Langevin thermostat (with a collision frequency of 1 ps^{-1}) and a Berendsen barostat at 300 K and 1 bar. Finally, 5 replicas of 200 ns were produced per starting conformation in the same conditions. They were analyzed using CPPTRAJ and the MMPBSA.py module from Amber20. Binding free energy calculations have been performed on the last 20 ns of each replica.

Supplementary Table 5. G4 structures used for molecular dynamics simulations.

PDB code	Topology	Sequence	Biological role
**1RDE**	Anti-parallel	5′GGTTGGTGTGGTTGG3′	Thrombin-binding DNA aptamer
**2M27**	Parallel	5′CGGGGCGGGCCTTGGGCGGGGT3′	Vascular endothelial growth factor promoter
**6O2L**	Parallel	5′TGAGGGTGGGTAGGGTGGGTAA3′	*c-MYC* gene promoter

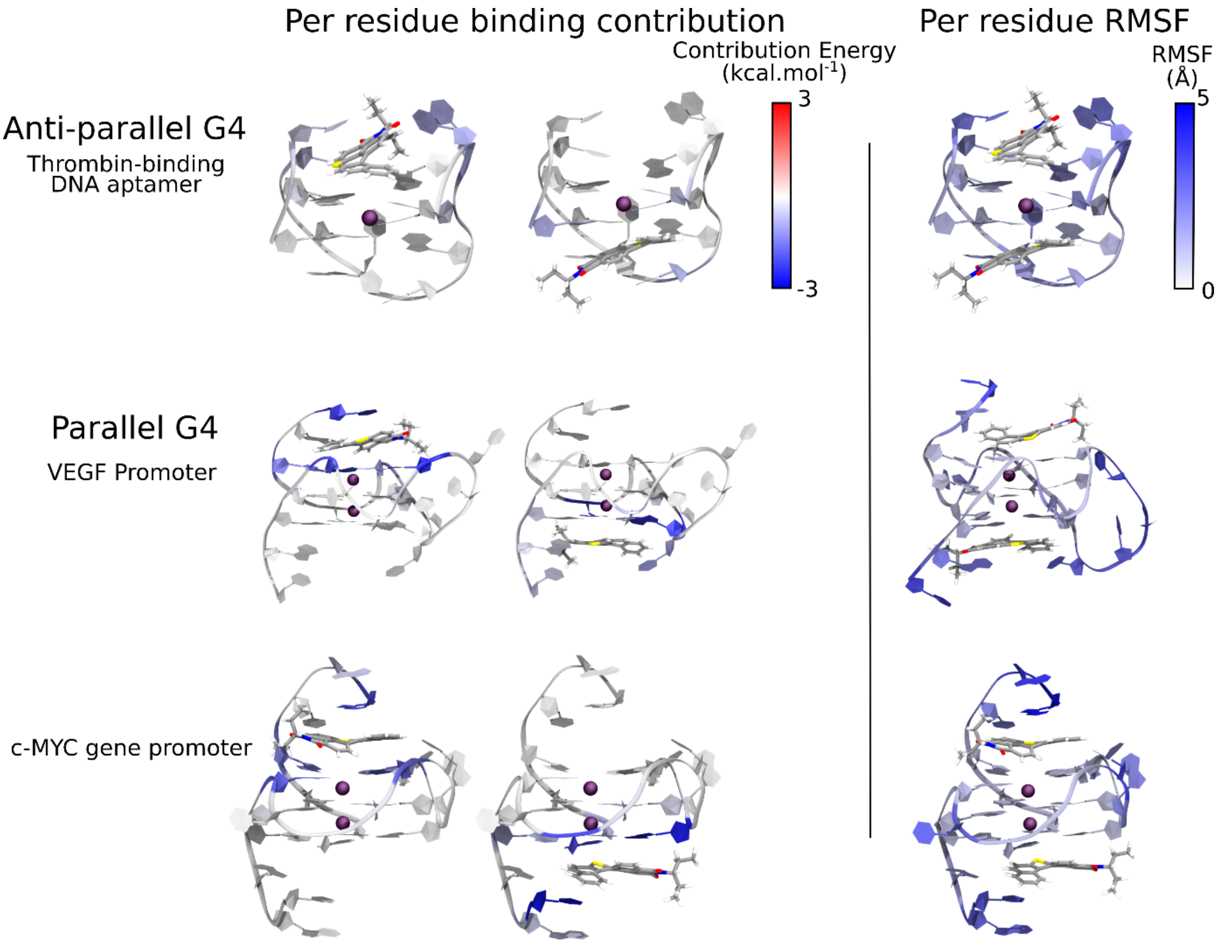

Supplementary Figure 18. Left: color map of the averaged per-residue contribution to the MMPBSA binding free energy for each ligand on the three G4s. Only the ligand involved in the calculated interaction is shown. Blue and red color indicate attractive and repulsive contributions respectively. Right: color map of root-mean-square fluctuation of each residue after alignment on the initial PDB structure.

Competitive binding assay between DBI and PhenDC3

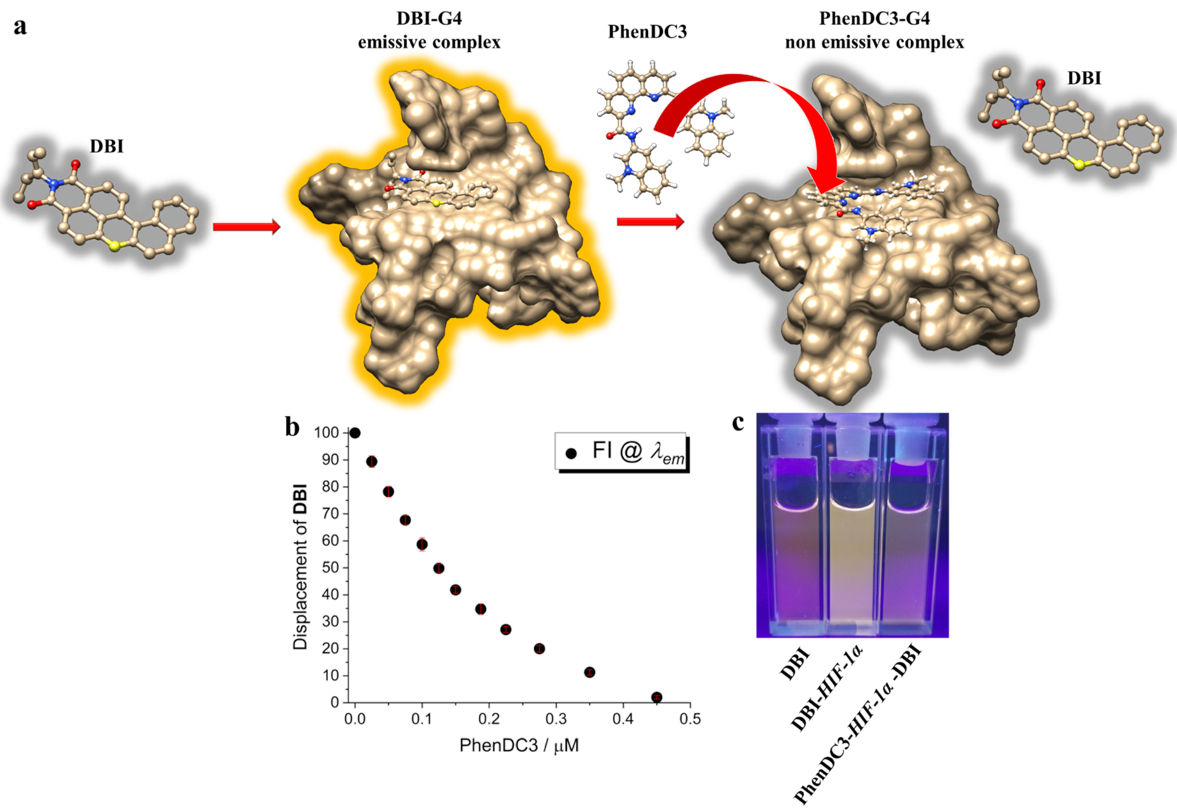

Supplementary Figure 19. a) Schematic representation of the competitive binding assay. **DBI** is weakly fluorescent in its unbound state. Upon binding to parallel G4s its emissive properties are enhanced. Competition for the same G4-binding site using the well-known G4-end stacker PhenDC3 causes the replacement of **DBI** from the G4 template and the resulting fluorescence quenching. **b)** Experimental evidence for the competitive binding between **DBI** and PhenDC3 (**DBI** = 1μM, *HIF-1α* = 1 μM, PhenDC3 = 0 to 0.45 μM, TRIS buffer = 50 mM, pH = 7.2 and KCl = 100 mM. **c)** Color changes detected upon UV-irradiation (312 nm) for **DBI**, **DBI**-*HIF-1α* complex and PhenDC3-*HIF-1α*-**DBI** mixture.

Extraction of exosomes from HeLa cells

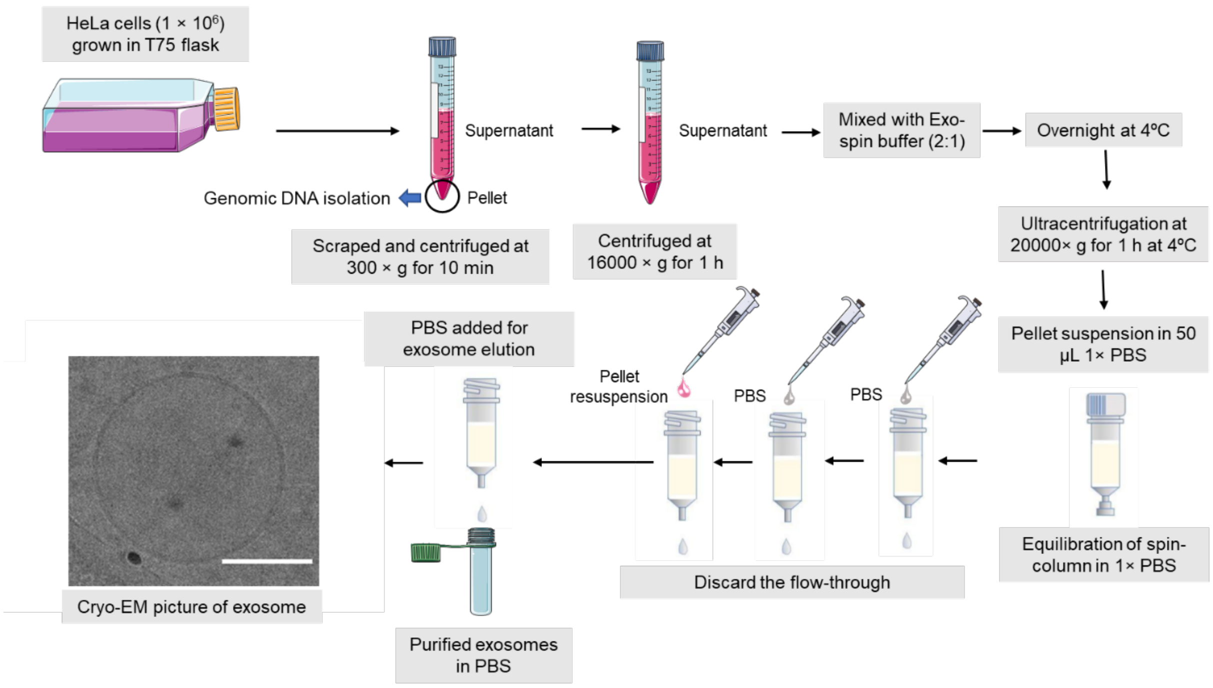

Supplementary Figure 20. Flow-chart showing step-by-step exosome isolation from HeLa cells. Cryo-EM image of the exosomes isolated from HeLa cells. Scale bar, 80 nm. Created by Biorender.com.

Proposed model for DBI-photoinduced DNA breaks

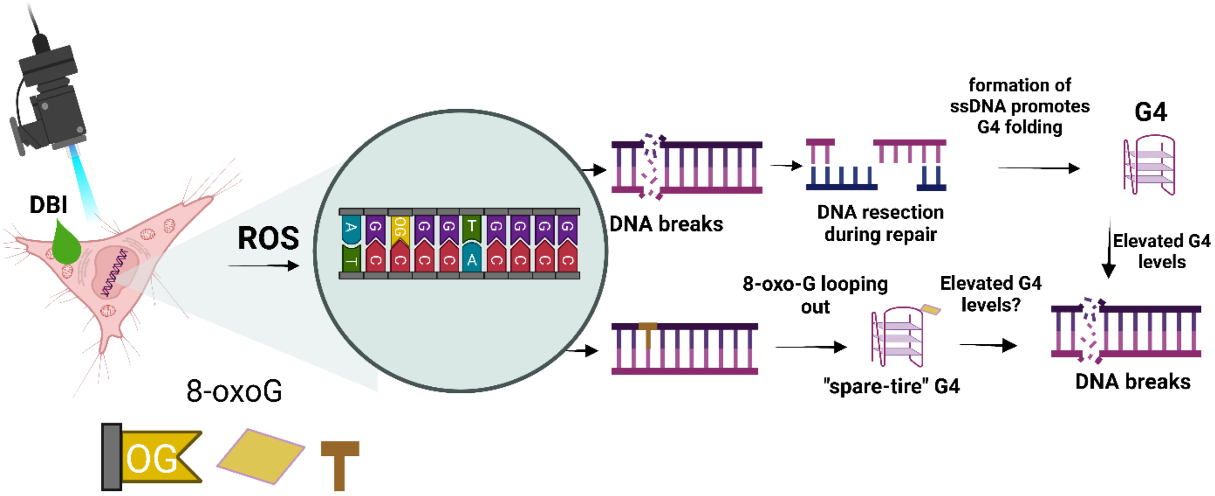

Supplementary Figure 21. Proposed model for **DBI**-photoinduced DNA breaks. Oxidative damage to DNA leads to DNA breaks through the formation of short ssDNA fragments that, if G-rich, can fold into G4 structures. In parallel, oxidatively damaged DNA can promote the formation of abasic sites which significantly impact the double helix stability. The oxidized guanine/s is/are well accommodated in the loops of G4 structures featuring extra G-runs "spare tire", therefore preserving the G4 folding. In both scenarios, the induction of G4s results in DNA breaks and thereby genome instability. Created by Biorender.com.

Local photo-activation of DBI in 24 hpf embryos

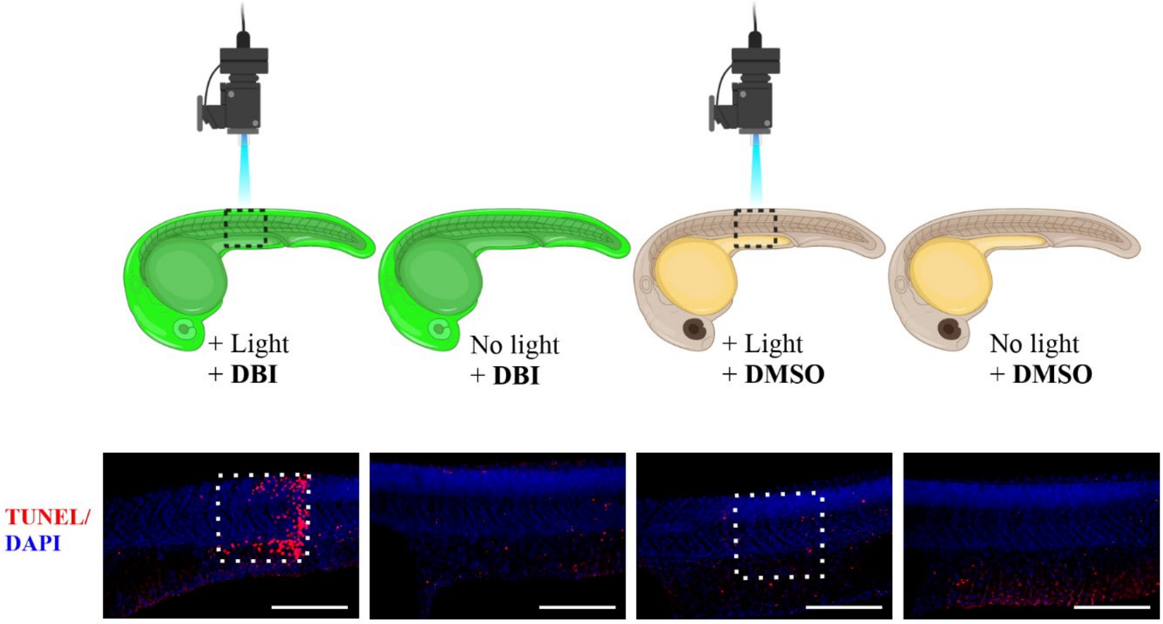

Supplementary Figure 22. Local photo-induction of **DBI**. Schematic illustration of local photo-activation of **DBI** in zebrafish embryos. The illustration was created by Biorender.com. The region of photo-irradiation is specified within the dotted lines using a Nikon A1 confocal 488 nm laser. Irradiated embryos without **DBI** and non-irradiated **DBI** treated embryos were used as controls. Co-localized signals are shown for DAPI (blue) and TUNEL (red). Scale bar: 150 µm.

Light-induced morphological changes on zebrafish embryos

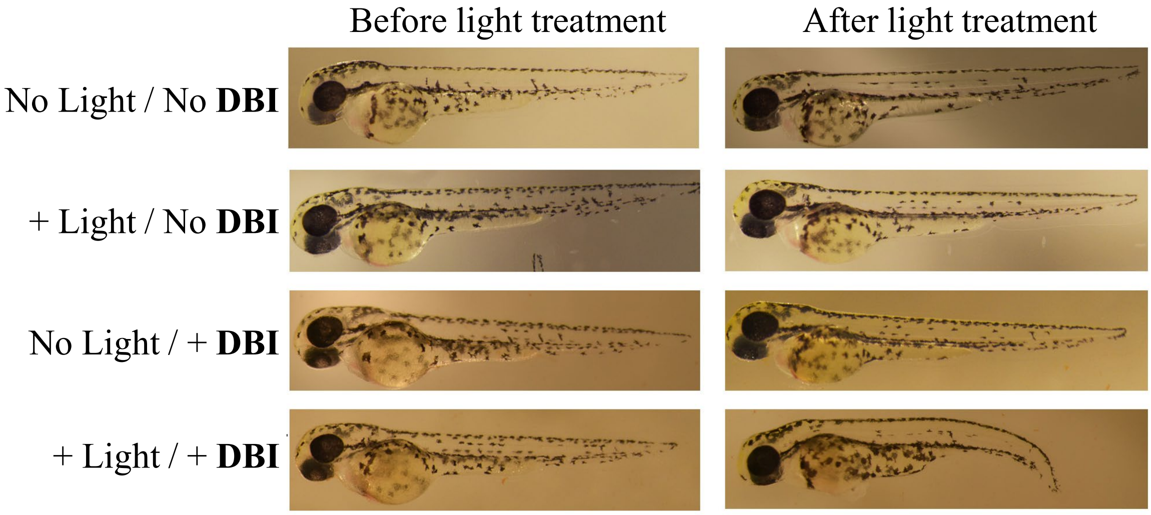

Supplementary Figure 23. Images of zebrafish embryos under different experimental conditions. Left-panels, zebrafish embryos before light treatment. Right-panels, zebrafish embryos after light treatment (55.6 mW cm^{-2} for 5 minutes) followed by 3 h of recovery in the dark. Zebrafish embryos were treated with **DBI** (10 µM) from 24 hpf to 48 hpf. Only +Light / +**DBI** condition (after light treatment) shows a clear morphological change occurring mostly at tail level.

Determination of the intracellular H_2O_2 level

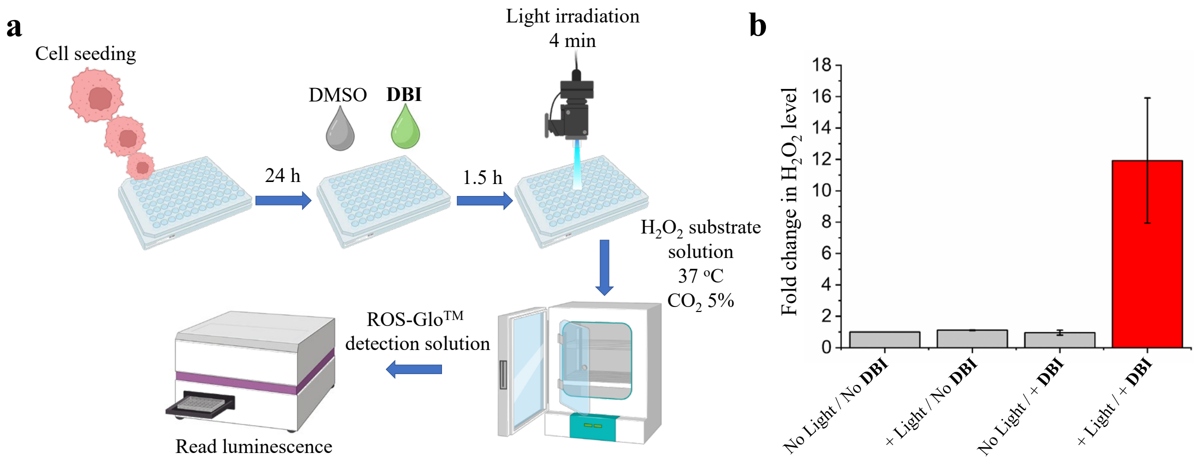

Supplementary Figure 24. a) Schematic representation of the bioluminescent assay used to measure the level of hydrogen peroxide (H_2O_2) directly in cell culture. Created by Biorender.com. **b)** H_2O_2 detection in HeLa cells treated with **DBI** (1 µM) or DMSO (0.02 % v/v). Where noted, the cells were irradiated with blue light using a LED light cube (30 mW cm^{-2}) for 4 min. After irradiation, ROS-Glo™ H_2O_2 kit (Promega) was used according to the manufacturer's instructions and the luminescence was recorded using a microplate reader. Data were normalized based on the experimental control conditions defined as: No Light / No **DBI**. Error bars indicate mean ± SD (n = 3).

EPR determination of radicals formed upon photoirradiation

2,2,6,6-tetramethyl-4-piperidine (TEMP) (Sigma-Aldrich) and DMPO (TCI chemicals) samples (5.10^{-3} M) were prepared in air atmosphere in capillary tubes, in chloroform and DMSO, respectively. In each The concentration of **DBI** was 10^{-4} M in all experiments. The irradiation performed using a Thorlab LED 530 nm, was directed into the EPR cavity while the spectrum was recorded. EPR assays were all carried out at room temperature using a Bruker E500 spectrometer operating at X-band (9.35GHz), sensitive cavity, with 100 KHz modulation frequency. The instrument settings were as follows: microwave power: 2-69 mW; modulation amplitude: 1 G; Hyperfine coupling constants a and g values were obtained with simulation of experimental spectra using easyspin (Matlab toolbox).

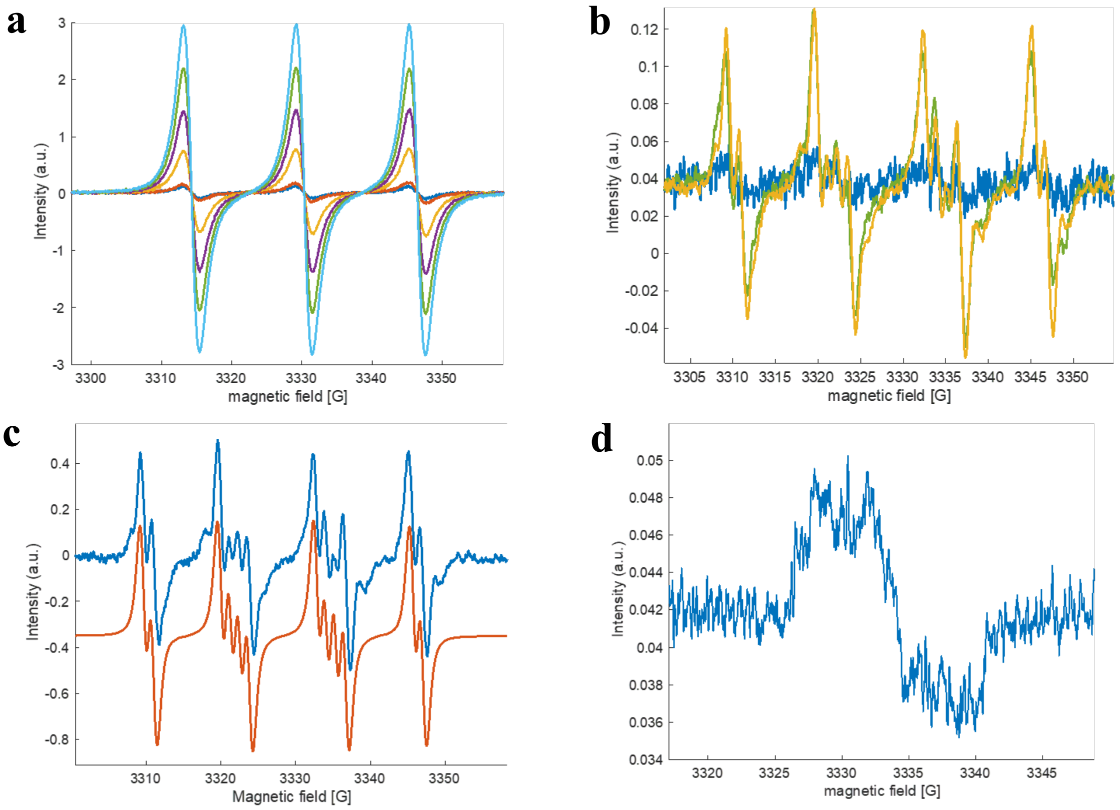

Supplementary Figure 25. a) EPR signal of a solution of **DBI** (10^{-4} M) and TEMP (5.10^{-3} M) in chloroform under 530 nm irradiation (dark blue: before irradiation; red: 30 s irradiation, orange: 3 min; purple: 6 min; green: 9 min; light blue: 12 min). Increasing TEMPO signal witnesses build up in $^1O_2$ concentration. **b)** EPR signal of a solution of **DBI** (10^{-4} M) and DMPO (5.10^{-3} M) in DMSO under 532 nm irradiation (blue: before irradiation; green: 3 min irradiation, orange: 14 min). **c)** Experimental (blue) and simulated (red) spectra of DMPO superoxide adduct obtained in (b). g = 2.006, aN = 12.85 G, aH = 10.35 G, aH = 1.28 G. **d) DBI** radical signature (10^{-4} M in DMSO) in the absence of spin trap. Intensity and resolution are too low to enable reliable simulation.

References

1. Frisch, M.J., Trucks, G.W., Schlegel, H.B., Scuseria, G.E., Robb, M.A., Cheeseman, J.R., Scalmani, G., Barone, V., Petersson, G.A., Nakatsuji, H. *et al.* (2016), Wallingford, CT.
2. Adamo, C. and Barone, V. (1999) Toward reliable density functional methods without adjustable parameters: The PBE0 model. *J. Chem. Phys.*, **110**, 6158-6170.
3. Galán, L.A., Andrés Castán, J.M., Dalinot, C., Marqués, P.S., Blanchard, P., Maury, O., Cabanetos, C., Le Bahers, T. and Monnereau, C. (2020) Theoretical and experimental investigation on the intersystem crossing kinetics in benzothioxanthene imide luminophores, and their dependence on substituent effects. *Physical Chemistry Chemical Physics*, **22**, 12373-12381.
4. Krishnan, R., Binkley, J.S., Seeger, R. and Pople, J.A. (1980) Self-consistent molecular orbital methods. XX. A basis set for correlated wave functions. *J. Chem. Phys.*, **72**, 650-654.
5. Tomasi, J. and Persico, M. (1994) Molecular Interactions in Solution: An Overview of Methods Based on Continuous Distributions of the Solvent. *Chem. Rev.*, **94**, 2027-2094.
6. Aidas, K., Angeli, C., Bak, K.L., Bakken, V., Bast, R., Boman, L., Christiansen, O., Cimiraglia, R., Coriani, S., Dahle, P. *et al.* (2014) The Dalton quantum chemistry program system. *Wiley Interdiscip. Rev. Comput. Mol. Sci.*, **4**, 269-284.
7. Jong, W.A.d., Harrison, R.J. and Dixon, D.A. (2001) Parallel Douglas–Kroll energy and gradients in NWChem: Estimating scalar relativistic effects using Douglas–Kroll contracted basis sets. *J. Chem. Phys.*, **114**, 48-53.
8. Nakajima, T. and Hirao, K. (2012) The Douglas–Kroll–Hess Approach. *Chem. Rev.*, **112**, 385-402.
9. Thordarson, P. (2011) Determining association constants from titration experiments in supramolecular chemistry. *Chem Soc Rev*, **40**, 1305-1323.
10. von Krbek, L.K.S., Schalley, C.A. and Thordarson, P. (2017) Assessing cooperativity in supramolecular systems. *Chem Soc Rev*, **46**, 2622-2637.
11. Liu, W., Lin, C., Wu, G., Dai, J., Chang, T.C. and Yang, D. (2019) Structures of 1:1 and 2:1 complexes of BMVC and MYC promoter G-quadruplex reveal a mechanism of ligand conformation adjustment for G4-recognition. *Nucleic Acids Res*, **47**, 11931-11942.
12. Ivani, I., Dans, P.D., Noy, A., Pérez, A., Faustino, I., Hospital, A., Walther, J., Andrio, P., Goñi, R., Balaceanu, A. *et al.* (2016) Parmbsc1: a refined force field for DNA simulations. *Nat Methods*, **13**, 55-58.
13. Wang, J., Wang, W., Kollman, P.A. and Case, D.A. (2006) Automatic atom type and bond type perception in molecular mechanical calculations. *J Mol Graph Model*, **25**, 247-260.
14. Bayly, C.I., Cieplak, P., Cornell, W. and Kollman, P.A. (1993) A well-behaved electrostatic potential based method using charge restraints for deriving atomic charges: the RESP model. *The Journal of Physical Chemistry*, **97**, 10269-10280.
15. Perdew, J.P., Ruzsinszky, A., Csonka, G.I., Vydrov, O.A., Scuseria, G.E., Constantin, L.A., Zhou, X. and Burke, K. (2008) Restoring the density-gradient expansion for exchange in solids and surfaces. *Phys Rev Lett*, **100**, 136406.
